# Layer-specific molecular signatures of colon anastomotic healing and leakage in mice

**DOI:** 10.1186/s10020-025-01167-9

**Published:** 2025-04-01

**Authors:** Hilal Sengul, Vasiliki Bantavi, Laura Gloeck, Andrew Y. F. Li Yim, Patrick Leven, Patrik Efferz, Bianca Schneiker, Mariola Lysson, Wouter J. De Jonge, Sven Wehner

**Affiliations:** 1 Department of Surgery, University Hospital of Bonn, 53105 Bonn, Germany; 2 Tytgat Institute for Liver and Intestinal Research, Amsterdam UMC - University of Amsterdam, 1105BK Amsterdam, The Netherlands; 3 Amsterdam Gastroenterology, Endocrinology, and Metabolism, Amsterdam UMC - University of Amsterdam, 1105BK Amsterdam, The Netherlands

**Keywords:** Colorectal anastomotic healing, Anastomotic leakage, Wound healing, Mucosal healing

## Abstract

**Background:**

Colon anastomotic leakage (CAL) is a postoperative complication originating from disturbed colon anastomotic healing (CAH). Wound healing involves several well-coordinated stages, which have not been comprehensively studied for CAH or CAL. This study aims to provide transcriptional profiles of different intestinal layers of anastomotic tissues throughout distinct healing stages and to identify CAL-related genes.

**Methods:**

Proximal colon anastomosis was constructed with 8 interrupted sutures in mice. Six hours, 24 h and 72 h after surgery, anastomotic complications were assessed. Transcriptional profiles of inner (mucosa and submucosa) and outer (muscularis externa) layer of the anastomotic and naive control tissues were analyzed with 3’ bulk mRNA sequencing to identify the layer-specific healing and leakage pathways. Selective target genes differing between CAL and CAH were measured for their protein expression.

**Results:**

Our data indicate that the mucosa/submucosa and muscularis externa enter inflammation stage at 6 h, proliferation stage at 24 h and tissue remodeling stage at 72 h during CAH. We observed that transcription profiles of the mucosa/submucosa, but not the muscularis externa, differ between CAH and CAL. Particularly, genes related to extracellular remodeling (including *Col18a1* and *Col16a1*) and wound healing (*Pdpn* and *Timp1*) showed lower expression in the mucosa/submucosa of CAL tissue compared to CAH. Conformingly, protein levels for collagens as well IL-34 were decreased in CAL, while the TGF-β-pseudo-receptor BAMBI was increased in CAL compared to CAH tissues.

**Conclusions:**

Mucosa/submucosa and muscularis externa are mostly in synchronization during the inflammation, proliferation, and extracellular remodeling stages during CAH. Transcriptional profiles within the anastomotic mucosa/submucosa differ between CAH and CAL in genes related to extracellular modelling and wound healing, indicating that genes of these pathways may contribute to CAL.

**Supplementary Information:**

The online version contains supplementary material available at 10.1186/s10020-025-01167-9.

## Introduction

After removal of a pathogenic entity during colorectal surgery, continuity of the intestine is restored by suturing the parts of the tissue, and this surgical side is called anastomosis (Lee et al. 2018; Nandakumar et al. 2009). Upon construction of an anastomosis, the tissue enters a complex healing program. However, in 1–19% of patients, colon anastomotic healing (CAH) is disturbed, which leads to leakage of the luminal content to the abdominal cavity resulting in abscess formation, peritonitis, or even sepsis (Su'a et al. 2017; Lee et al. 2018). This serious complication is known as colon anastomotic leakage (CAL) and is associated with high morbidity and mortality rates, increased rates of cancer recurrence, decreased quality of life (Snijders et al. 2012; Ramphal et al. 2018) and a high medico-economic burden (Flor-Lorente et al. 2023; Hammond et al. 2014).

CAL rates have remained similar in the last decades despite the improvement in surgical techniques and in perioperative management (Shogan et al. 2013; Chadi et al. 2016; Kelly et al. 2014; Bosmans et al. 2015; Lee et al. 2018), suggesting that the cause of CAL cannot be only attributed to errors in surgical techniques (Alverdy und Schardey 2021). Hence, in recent years extensive research has been conducted to identify the biological risk factors and to prevent/minimize the consequences of CAL. However, these attempts have not been successful as the mechanisms of CAH are still not well understood (Bosmans et al. 2015; Lam et al. 2020).

The wound healing process is categorized into three phases based on the main processes taking place in the wound: inflammation, proliferation, and remodeling (Morgan und Shogan 2022; Lam et al. 2020; Lundy 2014; Thompson et al. 2006). These stages have been well described in cutaneous wound healing and our current knowledge in CAH has been extrapolated from skin studies (Bosmans et al. 2015; Chadi et al. 2016; Lam et al. 2020; Lee et al. 2018; Lundy 2014). However, significant differences between skin and gut wound healing have been recognized (Bosmans et al. 2015; Chadi et al. 2016; Morgan und Shogan 2022). Additionally, factors regulating CAH stages might be colon-specific and can only be identified by analyzing the colonic anastomotic tissues.

To this end, an important factor to consider in CAH is the architecture of the intestinal wall consisting of four main layers: mucosa, submucosa, muscularis propria and serosa (Bosmans et al. 2015). As anastomotic surgery induces an injury in all layers, all of them undergo a healing process for a successful CAH, but only a few studies focused on the individual local roles of the different layers in the healing process. The mucosa layer for example contains the mucus-producing epithelium, the lamina propria and the muscularis, and mice lacking a functional mucus layer develop CAL (Bosmans et al. 2017). Moreover, recent studies showed that mucosa-microbiome interaction is one of the factors in CAL development, which further highlights its importance for successful healing (Lee et al. 2018; Shogan et al. 2016; Hajjar et al. 2023; Bachmann et al. 2022; Shi et al. 2022).The underlying submucosa contains large amount of collagens, and has the greatest tensile strength of the four layers (Thompson et al. 2006; Lundy 2014; Rosendorf et al. 2022) and is thought to be the source of strength in anastomotic tissue (Thompson et al. 2006; Lam et al. 2020; Rosendorf et al. 2022). The muscularis propria consists of mainly smooth muscle cells within a network of collagens (Thompson et al. 2006) but also contains immune and neuronal cell types (Schepper et al. 2018; Mogor et al. 2021), while the serosa contains a thin layer mesothelial cells covering the muscularis externa (Daams et al. 2013; Thompson et al. 2006). Overall, previous research indicates that these layers have individual cellular compositions, fulfil different roles and might be differentially regulated during CAH. However, a comprehensive analysis of the different layers during CAH and in CAL has not been conducted before except for the serosal layer, which cells have recently been identified to be part of the healing anastomotic environment (Weber et al. 2024).

To study the tissue specific transcriptional changes behind CAH and CAL (Bosmans et al. 2015; Alverdy und Schardey 2021), we analyzed separate layers (mucosa and adherent submucosa (M/SM) and muscularis externa (ME)) of the anastomotic tissues at 6 h, 24 h and 72 h after surgery in a colon anastomosis mouse model (Bachmann et al. 2020). Our data separate distinct layer-specific transcriptional responses during CAL and CAH and conclude that only the transcriptional profile of the M/SM can distinguish CAL from CAH tissue, directing future biomarker finding for CAL detection to this layer.

## Materials and methods

### Mice

Eight- to ten-week-old, female C57BL/6 J mice were purchased from Janvier (Saint Berthevin cedex, France) and maintained under SPF-conditions with a 12 h dark/light illumination cycle, temperature of 20–25 °C, humidity of 45–65%, standard rodent food and tap water ad libitum. State Agency for Nature, Environment and Consumer Protection (LANUV) approved the experiments (#81–02.04.2020.A098). Notably, all experiments were performed in female mice, which were used due to their less aggressive behavior compare to male mice. Mice aggression includes threating, chasing, and biting which all can cause skin sutures detachment and which comes along with significant stress, the latter known to also affect wound healing and intestinal inflammation responses.

### Anastomosis model

Mice were subjected to a previously established anastomosis model, which we adopted from Pommergaard et al. (2014). Mice were anesthetized by inhalation of isoflurane (2%, 3–5 L/min flow), and 10 mg/kg tramadol (Tralieve, Dechra, Aulendorf, Germany) was administered via s.c. injection 15 min before surgery. After median laparotomy, the cecum and proximal colon were located. Proximal colon (approximately 0.5 cm away from the cecum), was cut and an end-to-end anastomosis was constructed with 8 interrupted sutures using 8–0 Vicryl sutures (Ethicon, V1027G). The colon was placed back, the peritoneum and abdomen were sutured with two independent sutures. After surgery, mice were inspected, and their well-being was scored twice per day. Drinking water was supplied with 1 mg/kg tramadol (Aliud Pharma, Laichlingen, Germany) until the mice were sacrificed.

### Anastomotic scoring

After surgery, mice 6 h (n = 6), 24 h (n = 5) and 72 h (n = 8) were sacrificed, and anastomotic complications were assessed based on an established scoring system (Bosmans et al. 2016; van Helsdingen et al. 2023) (Figure S1).

### Tissue handling

Anastomotic tissues and additional naive proximal colon tissues from non-operated control mice (n = 5) were collected. They were cut open longitudinally and pinned to an agar covered plate. Pre-cooled, oxygenated Krebs–Henseleit buffer (126 mM NaCl; 2.5 mM KCl: 25 mM NaHCO3; 1.2 mM NaH2PO4; 1.2 mM MgCl2; 2.5 mM CaCl2, 100 IU/ml Pen, 100 IU/ml Strep and 2.5 μg/ml Amphotericin) was used to wash the tissues to remove feces. Then the buffer was removed and RNAlater (Thermo Scientific, AM7020) was poured over the tissues. Using forceps, the mucosa and submucosa (M/SM) were separated from and muscularis externa including serosa (ME) (Figure S2).

### RNA isolation

Total RNA was isolated from the samples using RNeasy Mini Kit (Qiagen, 74106) based on the manufacturer’s instructions. For homogenization step, Precellys homogenizer (Bertin Technologies) was used at 5000 RPM for 30 s. The samples were kept on ice throughout the extraction process. RNA concentrations were measured using the NanoDrop 1000 spectrophotometer (Thermo Scientific) and quality was assessed using the Agilent 2200 Tapestation. RNA samples with an RNA Integrity Number (RIN) score of ≥ 7 were analyzed with bulk mRNA sequencing.

### Bulk mRNA sequencing, data analysis and visualization

RNA-Seq libraries were prepared with QuantSeq 3’-mRNA Library Prep kit (Lexogen, Greenland, NH, USA) by the Genomics Core Facility of the University Hospital Bonn. Barcoded samples were sequenced on the Illumina HiSeq 2500 Instrument (50 bp single-end, 10 M reads). Raw data from the RNA-Seq was analyzed with “Partek Flow” software (Lexogen pipeline 12,112,017). The pipeline trimmed unique molecular identifiers (UMIs) and adapter sequences, aligned the reads with star2.5.3 and counted the genes with FeatureCounting. Ensemble transcripts release 99 for mm10 mouse alignment was used as refence genome. Unnormalized counts were downloaded for the subsequent bioinformatics analyses.

### Immunofluorescence staining

Anastomotic tissues were collected at POD1 and POD3, washed with ice-cold Krebs–Henseleit buffer, fixed with 4% PFA overnight followed by overnight incubation in 30% sucrose at 4 ℃. Tissues were then OCT embedded using TissueTek (Sakura, 4583) and cryosectioned in 10 um thickness. Tissue slides were then washed with PBS, permeabilized with 0.2% Ecosurf (PanReac AppliChem, A9779) for 10 min and blocked for 1 h with PBS containing 1% donkey serum and 0.1% Ecosurf. Sections were then stained with the primary antibodies Ly6G (Biolegend, 127602) in 1:100 and Iba-1 (Synaptic Systems, 234009) in 1:500 dilution at 4 ℃ overnight. Following the overnight incubation, tissue sections were washed with PBS three times and stained for the secondary antibodies (AF647, Biozol and AF488, Invitrogen) for 2 h at RT in dark. Sections were lastly stained with DAPI (1:1000) and mounted with ImmunoMount (Immunochemistry Technologies, AR-6516–01) and covered with glass coverslip. Microscopy images were obtained using and NIKON Ti-E Eclipse microscope. Microscopy and images were performed by the Nikon AR software.

### Histological staining and collagen quantification

Anastomosis were removed including 3 mm adjacent tissue on each side and were fixed in 4% PFA overnight, followed by washing and storage in 70% Isopropanol and subsequent embedding in paraffin. 5 µm slices were cut and stained using Masson Goldner Trichrome (MGT) staining kit (Morphisto, 12043) according to manufacturer instruction. Microscopy images were obtained using a NIKON Ti-E Eclipse microscope.

For quantification of collagen content, the MGT images were analysed using the software quPath (Version 0.5.1 (Bankhead et al. 2017)). The embedded Pixelclassifier module was trained using multiple annotations of exemplary regions (Collagen for green stained collagen areas, Tissue/Other for red stained muscle, tissue and brown stained nuclei, ignore* for background). The following parameters were used. Classifier: Random trees (RTrees), Resolution: Full (0.29 µm/px) and default multiscale features. Images were analysed by a blinded operator.

### ELISA

Anastomotic tissue was snap-frozen in liquid nitrogen and tissue lysates were prepared. Briefly, tissue was lysed with 1xRIPA buffer for 20 min, sonicated for 10 s and centrifuged at 4 °C for 10 min at maximum speed. Supernatants were collected and samples were stored at − 80 °C until usage. Protein concentrations were determined with a BCA kit (Thermo Scientific).

IL-34 (R&D Systems, DY5195-05), BAMBI/NMA (antibodies.com, A326426) and CCN1 (Abcam, ab253223) ELISAs were used according to the manufacturer's instructions. Total protein concentration of 100 µg was used for IL-34 and CCN1, and 50 µg for BAMBI/NMA measurement.

### Statistical analysis and data visualization

Differential gene expression (DEG) analysis of the annotated raw data was performed using Bioconductor package DESeq2 (1.40.2) using Rstudio (2023.06.0, Build 421). M/SM and ME layers were analyzed separately. Comparisons were made between different time points, and between CAH and CAL samples using the contrast function, where after the Wald statistic and the associated P values were calculated. To identify differentially expressed genes (DEGs) in a statistically robust way, we applied the Benjamini-Hochberg (BH) correction and used a padj < 0.05 threshold to control the false discovery rate (FDR). A log-fold change threshold is not concerned with the error rate, as it doesn't account for the variability of the expression values. We did not apply an LFC cut-off because our goal was to identify biologically relevant pathways involved in wound healing. Therefore, we included all significantly altered genes (padj < 0.05) in our analysis.

Only coding genes were included in the analysis. ClusterProfiler (4.8.2) (Wu et al. 2021; Yu et al. 2012) was used for gene set enrichment (GSE) and GeneOntology (GO) analysis. ggplot2 (3.4.3), DOSE (3.26.1) (Yu et al. 2015), tidyverse (2.0.0) (Wickham et al. 2019), AnnotationDbi (1.62.2), org.Mm.eg.db (3.17.0), forcats (1.0.0), RColorBrewer (1.1–3) and pheatmap (1.0.12) packages were used to visualize the data. Bar graphs were generated using Graphpad Prism version 10.1.2 (324) software. For protein and collagen expression statistical analysis was performed with Prism 10.0 using Student’s t test or one-way ANOVA. The values are expressed as mean ± SEM (standard error of mean). The significance levels were indicated as p ≤ 0.05 (*), p ≤ 0.01 (**), and p ≤ 0.001 (***).

## Results

### Validation of successful intestinal layer separation

**Fig. 1:**
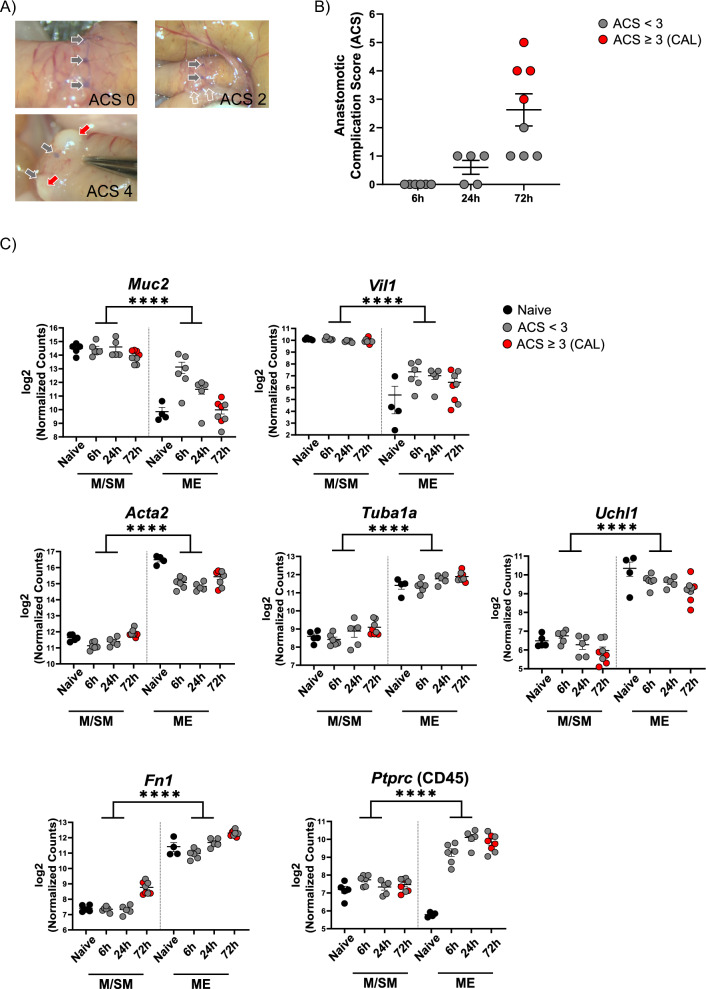
ACS of the mice and analysis of layer separation efficiency. Mice underwent anastomotic surgery and were euthanized at different postoperative time point. **A** Representative images for anastomotic complication score (ACS) 0 (left-top), ACS 2 (right-top) and ACS 4 (bottom left). Arrows point towards: single sutures (grey) and abscess (red) and adhesions (clear). **B** ACS of the mice included in this study. **C** Comparison of the expression level of the M/SM- and ME-specific genes to determine quality of layer-separation procedure. n = 4–8, Wald test with Benjamin-Hochberg correction, ****padj < 0.0001

To analyze the healing processes at different time points, we used a mouse model in which an end-to-end proximal colon anastomosis was constructed. 6 h, 24 h and 72 h after surgery, we sacrificed the mice and assessed the anastomotic complications based on a scoring system established before (Bosmans et al. 2016; van Helsdingen et al. 2023) (Figure S1). At 6 h and 24 h, the anastomotic complication score (ACS) was 1–2, indicating no observable anastomotic defects (Fig. 1A + B). At 72 h, 50% of the mice (n = 4) showed ACS 1 or 2 and 50% (n = 4) had ACS 3 or 4 which corresponds to anastomotic defect (Fig. 1A + B). Since ACS ≥ 3 indicates insufficient wound healing, hereafter, the mice with ACS 3–4 will be referred to as CAL.

Next, we separated the mucosa and submucosa (M/SM) layer of the anastomotic tissues from the muscularis externa and serosa (ME) layer (Figure S2). As control samples, we obtained M/SM and ME from non-operated naive proximal colon. After performing 3’ bulk mRNA sequencing, we first assessed whether M/SM and ME were separated successfully. The M/SM-specific genes *Muc2* and *Vil1* were significantly higher in the M/SM compared to the ME layer. In contrast, *Acta2* (smooth muscle cell marker (Chen et al. 2023), *Tuba1a* (encodes for alpha tubulin (Aiken et al. 2017)) and *Uchl1* (enteric neuron marker PGP9.5 (Blair et al. 2012)) expression were significantly higher in the ME compared to the M/SM (Fig. 1C). Although detectable levels of *Muc2* and *Vil1* within the ME samples might indicate minor cross-contamination, their difference between the layers were significant indicating that the layer separation was successful. Interestingly, although the granulation tissue developing at the anastomotic site spans both layers in vivo, it remained mainly attached to the ME (Figure S2) after mechanical separation. In line, we detected higher levels of *Fn1* (fibronectin marker), which plays a role in granulation tissue formation (Lenselink 2015), and *Ptprc* (CD45, a pan-immune cell marker) transcripts in the ME compared to M/SM (Fig. 1C).

### M/SM and ME initiate inflammation and angiogenesis 6 h after surgery

**Fig. 2:**
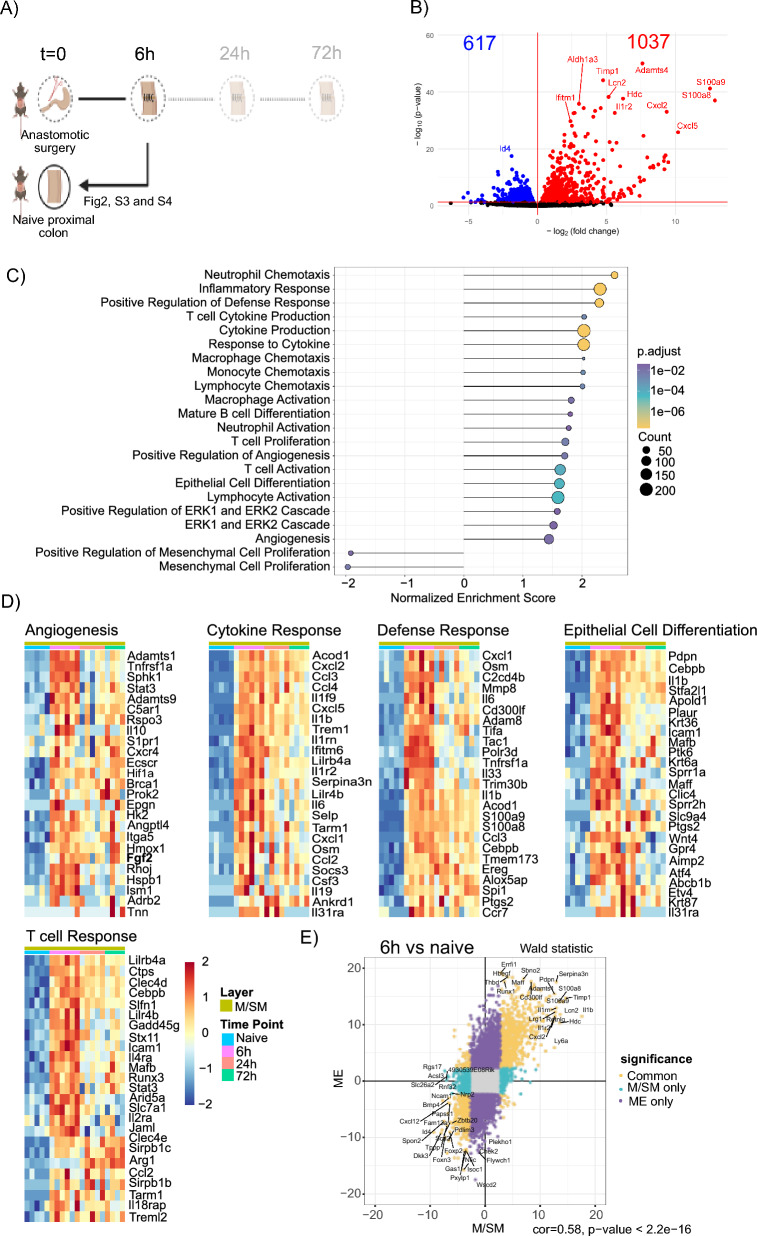
Gene expression of the M/SM of the anastomotic tissue at 6 h compared to naive controls. **A** Experimental setup. Direction of the arrow indicates the comparisons made **B** Volcano plot of DEGs (red and blue shows up-/downregulated genes, respectively) in the M/SM layer of anastomotic tissue collected at 6 h compared to naive tissue **C** Selected significantly enriched pathways in the M/SM layer of anastomotic tissue at 6 h compared to the naive tissue based on gene set enrichment analysis (GSEA) **D** Heatmap showing the expression pattern of the genes associated with the indicated pathways in the M/SM of naive and anastomotic tissues collected at 6 h, 24 h and 72 h. **E** Correlation plot based on the Wald statistics of the genes shared between M/SM and ME layer at 6 h after surgery compared to their corresponding naive controls

To achieve a longitudinal transcriptional analysis of anastomotic healing, we performed comparisons between different time points. We started by comparing the anastomotic tissues collected at 6 h to the naive condition to identify the very first pathways activated after surgery (Fig. 2A). Afterwards, we compared 24 h to 6 h and 72 h to 24 h time points.

A comparison of M/SM at 6 h and naive samples identified 1037 and 617 genes as significantly upregulated and downregulated at 6 h, respectively (Fig. 2B). Gene set enrichment analysis (GSEA) showed that the most prominently induced pathways 6 h post-surgery were related to inflammation, including neutrophil and macrophage chemotaxis, cytokine response and positive regulation of bacterial defense (Fig. 2C** + **D). Interestingly, besides the expected innate immune response, our data indicate that adaptive immune responses, e.g. enrichment of the lymphocyte (T and B cell) associated gene sets (Fig. 2C) as well as the interferon response (Figure S3A), are also upregulated early on in CAH.

Vascularization is considered as crucial for successful healing and research shows that hypoxia followed by devascularization causes CAL in mice (Miltschitzky et al. 2021; Shogan et al. 2016). Interestingly, our data show that angiogenesis pathways were enriched, and *HIF1α* and *FGF2*, which induces angiogenesis upon activation of HIF1α signaling (Conte et al. 2008), were upregulated 6 h after surgery (Fig. 2D). Additional pathways associated with vascular development including vascular endothelial growth factors (VEGF) production and VEGF regulation were also enriched in the M/SM. Interestingly, even though VEGF production is a downstream effect of HIF1 activation, we did not observe an upregulation of HIF1 target genes in the early stages (6 h and 24 h) of the healing process, neither in the M/SM nor the ME layer (Figure S3B-C, respectively). Other pathways that were altered at the 6 h time point included enrichment of epithelial cell differentiation and reduction in mesenchymal cell proliferation (Fig. 2C + D). Overall, these data show that although the most prominent defining feature of the 6 h time point is inflammation, the tissue upregulates several wound healing related pathways simultaneously.

We next turned our attention to the transcriptional changes in the separated ME layer. Interestingly, compared to the naive counterpart, the number of significantly regulated genes in ME collected 6 h after surgery was much higher (3530 and 3213 significantly up-/downregulated genes, respectively) than that of M/SM. This can be explained by the presence of the granulation tissue, which predominantly remained in ME after the mechanical layer separation. A comparison of the expression levels of the genes detected in both M/SM and ME showed that the transcriptional profiles of the M/SM and ME positively correlate (Fig. 2E). In line with this, GSEA indicated that ME was also strongly enriched in innate and adaptive immune cell response, angiogenesis, and defense response 6 h after surgery (Figure S4B). These findings show that transcriptional pathways in the ME and M/SM overlap substantially in the early postoperative and more pronounced in the granulation-tissue containing ME.

### Proliferation phase starts 24 h after surgery

**Fig. 3:**
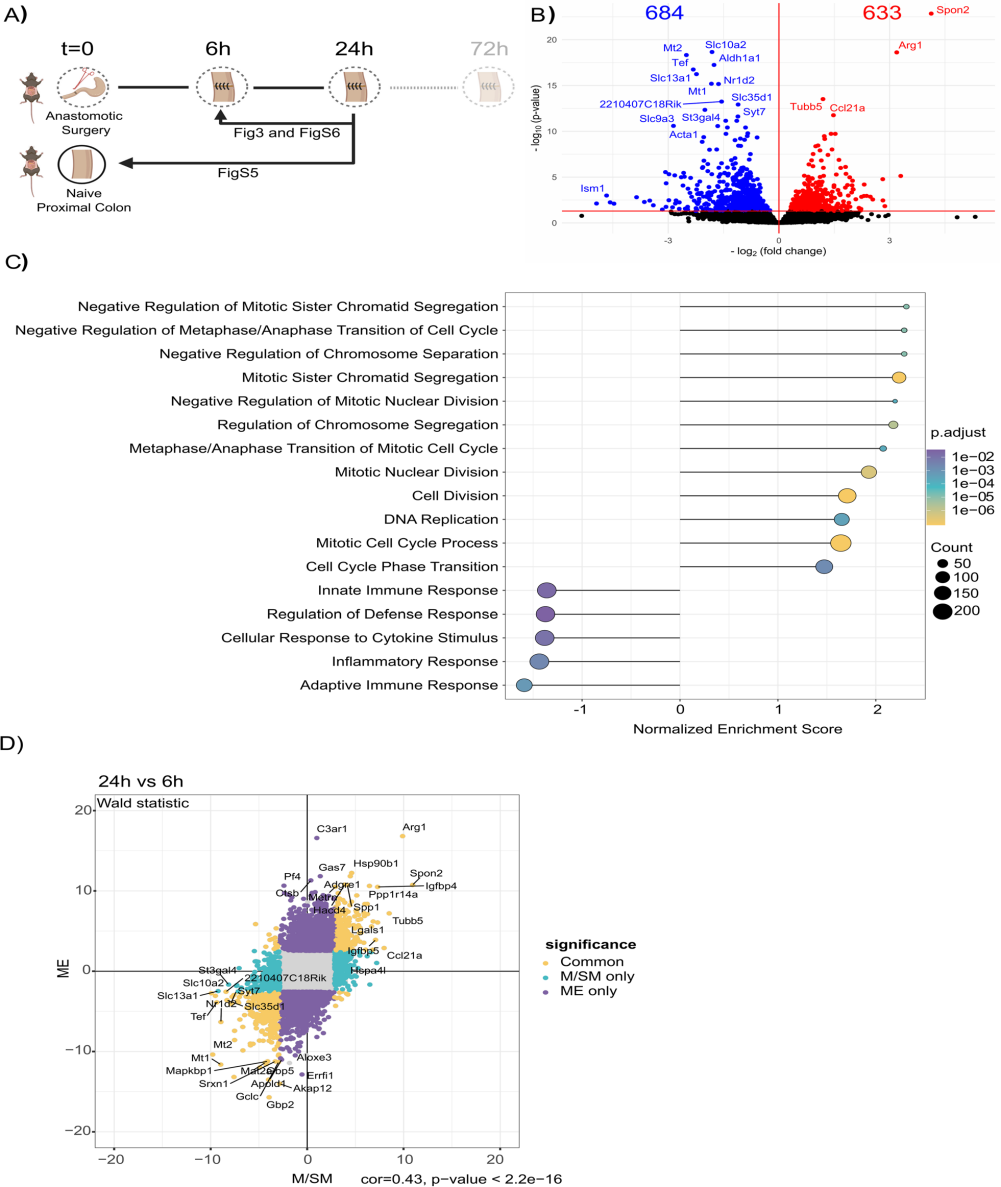
Gene expression of the M/SM layer of the anastomotic tissue at 24 h compared to 6 h. **A** Experimental setup. Direction of the arrows indicate the comparisons made in this section **B** Volcano plot of DEGs (red and blue shows up-/downregulated genes, respectively) in M/SM layer of anastomotic tissue collected at 24 h compared to 6 h. **C** Selected significantly enriched pathways in the M/SM layer of anastomotic tissue at 24 h compared to 6 h based on GSEA. **D** Correlation plot based on the Wald statistics of the genes shared between M/SM and ME layer at 24 h after surgery compared to 6 h

We then characterized the transcriptional changes between the 6 h and 24 h time points (Fig. 3A). In the M/SM, we detected 633 and 684 genes that were significantly up-/downregulated at 24 h, respectively (Fig. 3B). GSEA revealed that pathways involved in cell proliferation such as regulation of chromosome segregation, nuclear division, DNA replication, and cell cycle transition were enriched at 24 h. Therefore, the 24 h time point can be characterized as the proliferation phase of the CAH (Fig. 3C). Interestingly, negative regulation of cell division was also significantly enriched (Fig. 3C), which might be indicative of cell specific regulation of proliferation.

We also observed a significant downregulation of the innate and adaptive immune responses, cytokine response and host defense at 24 h time point. Although these pathways were still positively enriched compared to naive tissue (Figure S5), they were downregulated compared to the 6 h time point, indicating that host´s immune response within the first 24 h is predominantly activated in the immediate postoperative phase.

Similar observations were made in the ME. We identified 2016 and 1767 significantly up-/downregulated genes at the 24 h compared to the 6 h (Figure S6A), respectively. Correlation analysis using the DEGs shared between M/SM and ME at 24 h compared to 6 h showed a significant positive correlation between these layers (Fig. 3D). In line with this, GSEA showed that ME also undergoes the proliferation stage at 24 h time point (Figure S6B), indicating that both layers move simultaneously through the stages of the wound healing. Interestingly as opposed to the M/SM, in the ME, inflammation-related pathways (e.g., leukocyte and lymphocyte proliferation, and adaptive immune response) were significantly enriched at 24 h (Figure S6B). These data indicate that the initial wound healing stages in the M/SM and the ME are mostly synchronized, except the inflammation-related pathways, which appeared to be significantly downregulated in the M/SM but upregulated in the ME.

### Extracellular matrix remodeling starts at 72 h after surgery

**Fig. 4:**
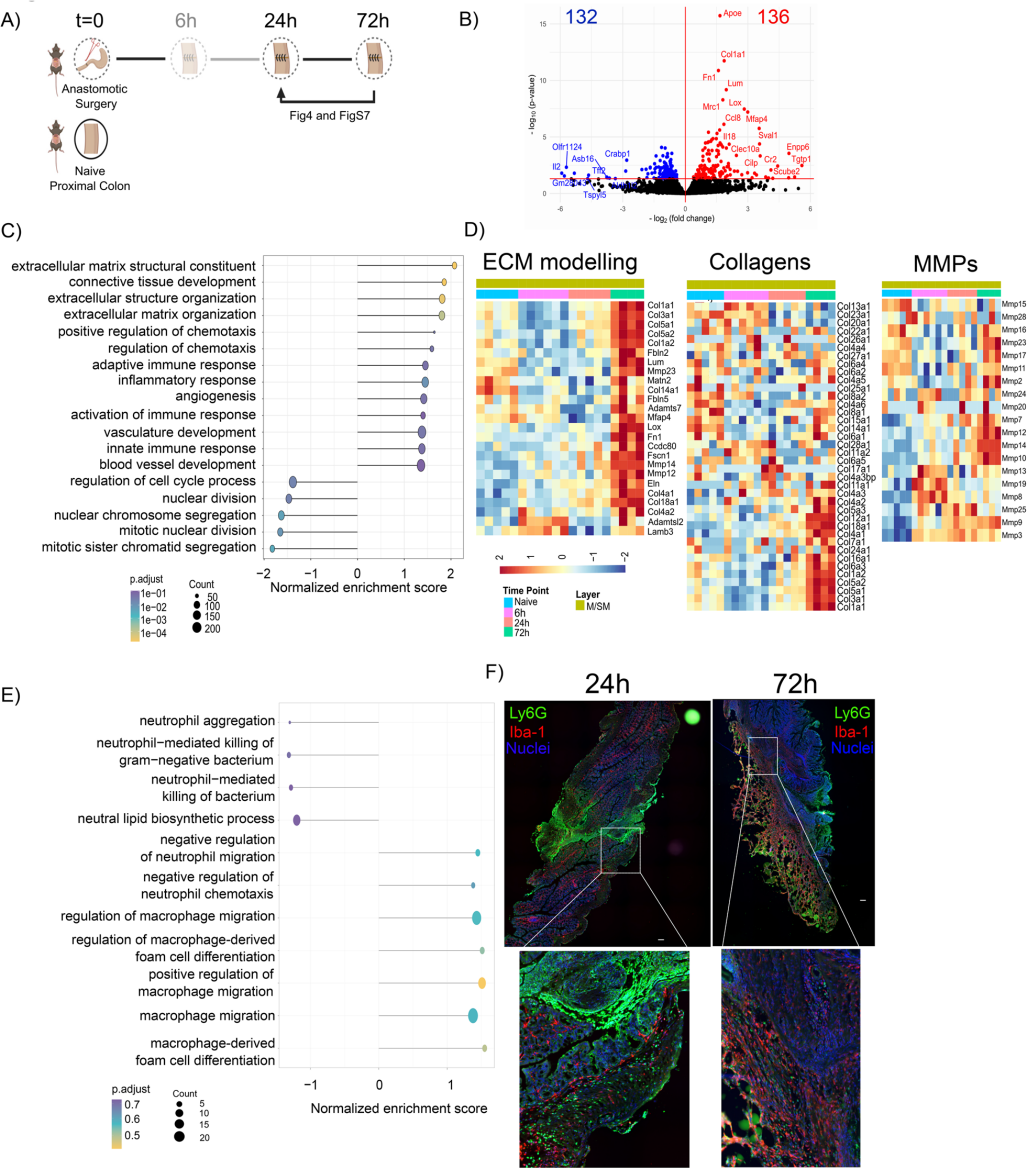
Gene expression of the M/SM layer of the anastomotic tissue at 24 h compared to 72 h. **A** Experimental setup. Direction of the arrow indicates the comparisons made in this section. **B** Volcano plot of DEGs (red and blue shows up-/downregulated genes, respectively) in the M/SM layer of anastomotic tissue collected at 72 h compared to 24 h. **C** Selected significantly pathways in M/SM the layer of anastomotic tissue at 72 h compared to 24 h based on GSEA. **D** Heatmap showing the expression pattern of genes related to ECM modelling, collagens and MMPs in the M/SM layer of naive and anastomotic samples collected at 6 h, 24 h and 72 h. **E** Pathways associated with macrophage and neutrophil function in the M/SM layer of anastomotic tissue at 72 h compared to 24 h based on GSEA. **F** Large images of immunofluorescence staining from colonic anastomosis at 24 and 72 h with corresponding high-resolution images of the indicated areas in the granulation tissue and muscularis externa: macrophages (IBA-1), neutrophils (Ly6G) and DAPI (Nuclei)

Finally, we investigated the healing pathways specific for 72 h time point in M/SM and ME of anastomotic tissues in comparison to the 24 h time point (Fig. 4A). Notably, at 72 h the clinical scoring identified two groups based on ACS (Fig. 1B). For the analysis of healing pathways, we focused on the group with ACS < 3 while the samples with ACS ≥ 3 were separately analyzed afterwards.

We identified 136 and 132 genes that were significantly up-/downregulated at 72 h compared to 24 h, respectively, in the anastomotic M/SM (Fig. 4B). GSEA showed an enrichment of extracellular matrix (ECM) remodeling related pathways (Fig. 4C). We observed that most genes associated with ECM remodeling reached their peak expression at 72 h suggesting that the remodeling phase starts at 72 h (Fig. 4D). Within the ME, 563 and 355 genes were significantly up-/downregulated at 72 h compared to the 24 h (Figure S7A). Correlation analysis based on the DEGs that are common in both M/SM and ME layers showed a significantly positive correlation (Figure S7B) and GSEA indicated that ECM remodeling-related pathways were also upregulated in ME (Figure S7C, D).

Matrix metalloproteinases (MMPs) and collagens are crucial components of ECM remodeling and their role in development of CAL has been previously reported. Hence, we analyzed their expression pattern throughout healing and observed that MMPs and collagens were produced in a time-dependent manner in both layers (Fig. 4D and Figure S7E). For instance, *MMP3* is upregulated at 6 h time point, and remains on a similar level throughout the healing. In contrast, *MMP16*, *MMP17* and *MMP23* were only upregulated at 72 h time point in both layers. Among the collagens we detected, *Col5a1*- *Col5a2*, *Col1a1*-*Col1*a2, *Col3a1* and *Col16a1* expression peaked at 72 h time point while *Col13a1* and *Col17a1* were upregulated only at 6 h time point in the M/SM and ME, respectively (Fig. 4D and Figure S7E).

Our GSEA also showed a significant enrichment in inflammatory and angiogenesis pathways at 72 h compared to 24 h in M/SM (Fig. 4C) suggesting that there might be a second wave of these pathways in the M/SM following the proliferation phase. This second “wave” of inflammation is characterized by an enrichment of gene expression related to macrophage function and migration, while neutrophil-related genes showed a negative enrichment score (Fig. 4E). Although p-adjusted value did not reach significance, immunofluorescence microscopy confirmed these findings showing a clear switch from a dominant Ly6G^+^ neutrophil infiltrate at 24 h towards IBA-1^+^ macrophage dominated cell infiltrate at 72 h (Fig. 4F). In the ME, the inflammation related pathways did not significantly differ between 72 and 24 h time point, while angiogenesis was enriched (Figure S7B).

Overall, these data present transcriptional changes of murine CAH in its initial stages. Both ME and M/SM went through different transcriptional processes mostly synchronously, with some differences in the duration of some processes. Inflammation, angiogenesis and defense response activated within the first 6 h of anastomotic surgery. The proliferation phase starts at 24 h and ECM remodeling starts around 72 h after surgery.

### Transcriptional signatures within the M/SM but not ME distinguish CAL from CAH

**Fig. 5:**
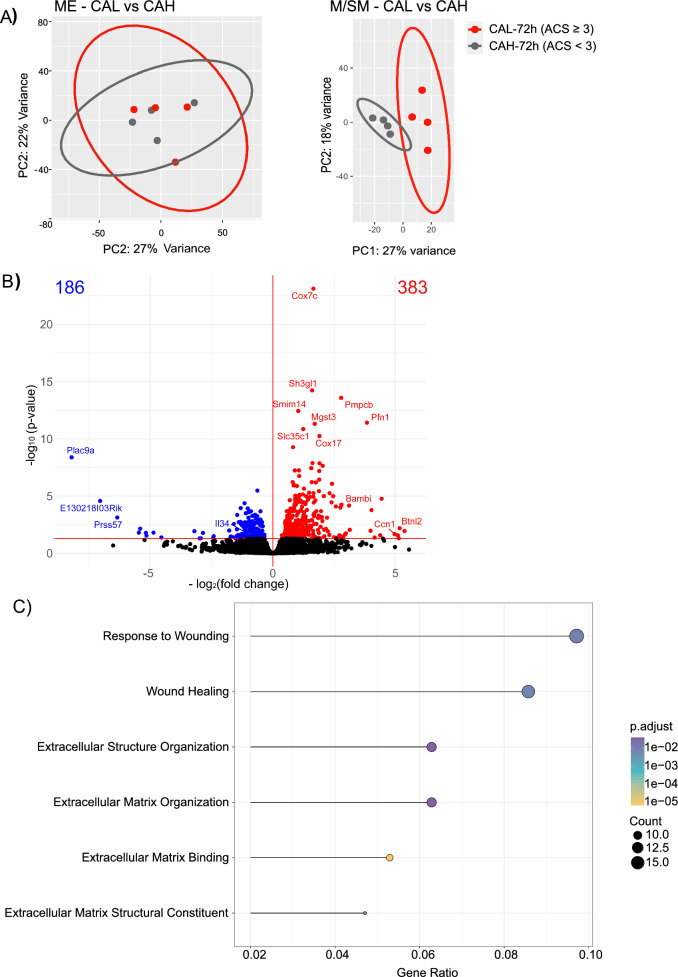
Transcriptome of the M/SM at 72 h distinguishes CAL and CAH. **A** Principal component analysis (PCA) of ME and M/SM in CAL and CAH **B** Volcano plot of DEGs (red and blue shows up-/downregulated genes, respectively) in the M/SM layer in CAL compared to CAH **C** GOterms associated with the significantly downregulated genes in CAL compared to CAH

As shown in Fig. 1, at 72 h, 50% of the mice developed CAL. Next, we compared the transcriptional profiles of CAH (ASC < 3) and CAL (ACS ≥ 3) samples collected at 72 h. Principal component analysis (PCA) showed that ME of the CAL and CAH samples clustered together (Fig. 5A), which was corroborated by the lack of any detectable DEGs between the CAL and CAH samples, indicating that CAH and CAL cannot be distinguished based on the ME´s transcriptional profile. In contrast, M/SM samples of CAH and CAL tissues clustered separately and DEG analysis showed 383 and 186 significantly up/-downregulated, respectively, in CAL (Fig. 5A + B).

### ECM remodeling and wound healing pathways are downregulated in CAL

We then analyzed the differentially regulated pathways between CAH and CAL at 72 h in their M/SM layers by performing Gene Ontology (GO) term analysis. Whilst the analysis of the upregulated CAL genes did not result in any enriched pathways, the downregulated genes in the CAL tissues were related to numerous GOterms. We then focused on top 25 terms most significant GOterms and observed that CAL-associated downregulated genes were associated with ECM remodeling and its components such as collagens, wound healing, and cell migration (Fig. 5C). Verifying the reduction in ECM remodeling process, GSEA showed that ECM component was downregulated in CAL (Figure S8).

**Fig. 6:**
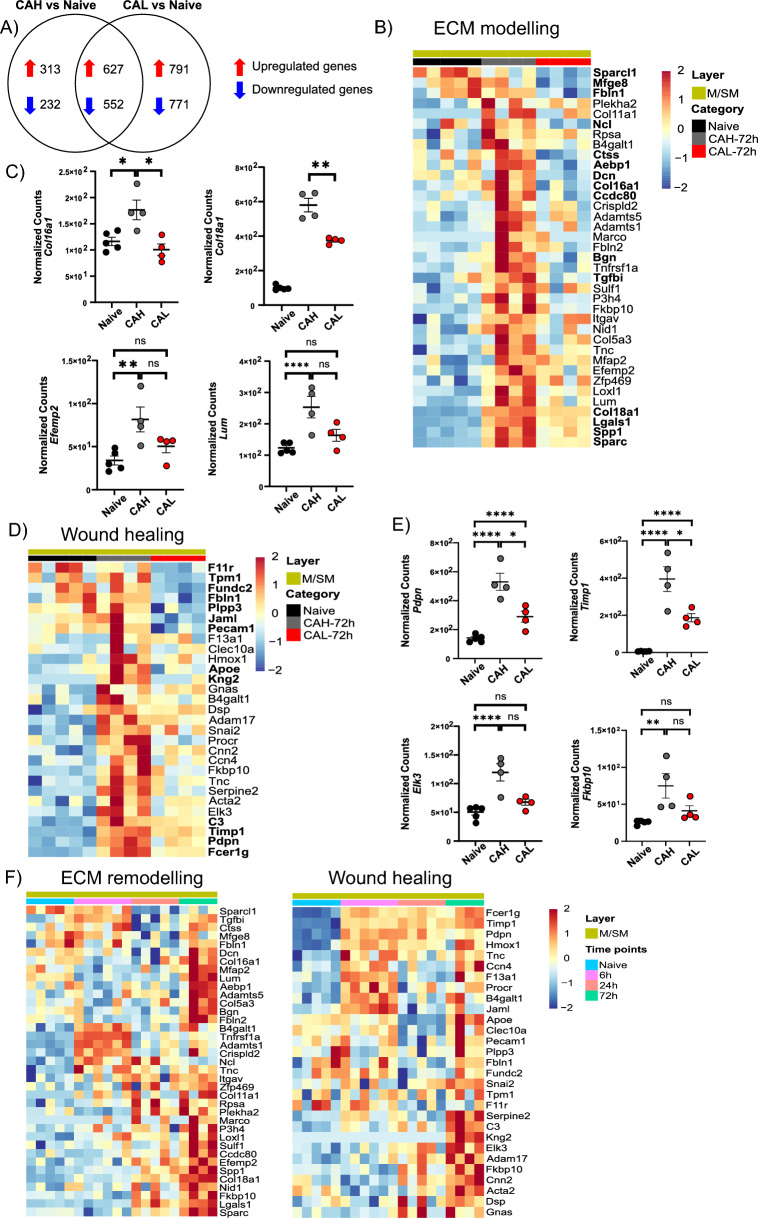
M/SM of CAL lacks certain ECM remodelling- and wound healing- associated gene expression. **A** Venn diagram showing the number of genes unique to and shared between CAH and CAL. **B** Heatmap showing the gene expression of CAL-associated genes involved in ECM-remodelling. **C** Bar plots showing differences in expression levels of Col16a1, Col18a1, Efemp2 and Lum in the M/SM layer of naïve, CAH and CAL tissue. **D** Heatmap showing the gene expression of CAL-associated genes involved in wound healing **E** Bar plots showing differences in expression levels of Pdpn, Timp1, Elk3 and Fkbp10 in the M/SM layer of naïve, CAH and CAL tissue. **F** Heatmap showing the expression pattern of CAL-associated genes in the M/SM layer of naive and anastomotic samples collected at 6 h, 24 h and 72 h. n = 4, Wald test with Benjamin-Hochberg correction, ****padj < 0.0001. ns: not significant; padj ≥ 0.05; *padj < 0.05; **padj < 0.01; ****padj < 0.0001

Of the downregulated pathways in CAL tissue, we focused on the two most relevant pathways in CAL development, namely wound healing, and ECM remodeling. To investigate if these pathways were only enriched in CAH and not in CAL tissue, we incorporated the M/SM of the naive colon into the analysis. Comparison of CAH and CAL samples with naive tissues showed that there were 627 and 552 significantly up-/downregulated genes, respectively, that are common between CAH and CAL (Fig. 6A). Our GOterm analysis showed that upregulated genes shared between CAH and CAL were related to wound healing, and ECM remodeling pathways (data not shown) suggesting that the M/SM of CAL also had these enriched these pathways, however, it lacked specific genes that are involved in these pathways.

We then proceeded to identify the genes that were enriched in CAH but not in CAL tissue. Our analysis showed 37 ECM remodeling-associated genes whose levels remained lower than and comparable to the naïve tissue in CAL, whereas their expression was higher in the CAH tissue compared to the naïve. Among these genes, 11 of them including *Fbln1*, *Col18a1* and *Col16a1* were significantly higher in the CAH compared to CAL (Fig. 6B** + **C). Analysis of wound healing-associated genes identified 29 genes with low levels in the CAL tissue and 15 of those, including *Pdpn* (gp38) (fibroblast marker), *Timp1* (MMP inhibitor), and *Fcer1g* (involved in anti-microbial immunity) was significantly different between CAH and CAL (Fig. 6D + E). We also followed the expression pattern of the CAL-associated genes throughout the healing process in the M/SM of the samples with ACS < 3 and observed that the expression of these genes exhibits different dynamics. Interestingly, the above-mentioned CAL-associated genes *Pdpn*, *Timp1 and Fcer1g* were upregulated as early as 6 h and maintained higher levels throughout the healing. On the other hand, Kng2, Serpine2, and C3 were upregulated specifically at 72 h (Fig. 6F).

**Fig. 7:**
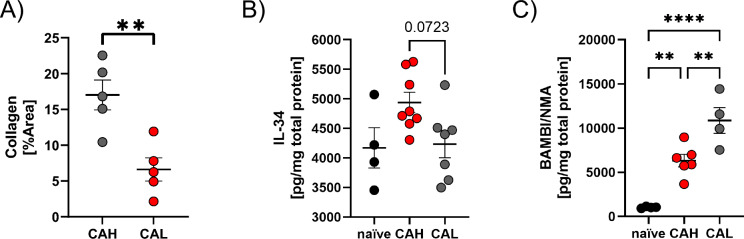
C57BL6/J mice underwent anastomotic surgery and anastomoses were harvested 72 h after surgery. **A** Cross sections of anastomosis were analyses for collagen contents in Masson Goldner Trichrome staining. Bar plots show the percentages of collagen area in cross sections from anastomoses of the CAL or CAH groups. Student’s t test, n = 5, ** = p < 0.01. **B + C** Bar plot showing concentration of (**B**) IL-34 or (**C**) BAMBI/NMA in anastomotic tissue or corresponding naïve tissue for naïve, CAL and CAH. n = 4–8, one-way ANOVA with Tukey correction. ** = p < 0.01; **** = p < 0.0001

Finally, we aimed to confirm some of the transcriptional changes on protein levels. Given that collagen genes were expressed higher in the CAH compared to the CAL groups, we validated these findings by a Masson Goldner Trichrome (MGT) staining and subsequent quantification of collagen contents by blinded and unbiased computational analyses. In tissues with CAL, the collagen content was significantly lower compared to CAHi (Fig. 7A). Furthermore, from the list of 569 DEGs between CAL and CAH (Fig. 5B), we identified those that were at least down- or upregulated with an log2 values of − 1 or + 1.5, respectively and with adjusted p-values of less than 0.05. From the resulting 31 genes (Supplemental Table 1) we manually screened the literature for their involvement in wound healing processes and availability of ELISA kits or validated antibodies for western blotting. Consequently, we tested lysates obtained from full thickness CAL and CAH anastomotic tissues as well as naïve proximal colon tissues for IL-34 (downregulated in CAL compared to CAH) as well as Ccn1, Btnl2 and the TGF-β decoy receptor Bambi (upregulated in CAL versus CAH). Ccn1 was neither detected in naïve nor anastomotic tissues, while Btnl2 was only detected in naïve samples but in none of the anastomotic tissues (results not shown). In line with our transcriptional data, IL-34 was upregulated in anastomotic versus naïve colon and showed trend in being reduced in CAL samples compared to CAH tissue (p = 0.07, Fig. 7B). In contrast, but also in line with the RNA levels, Bambi was induced in anastomotic tissues compared to naïve samples and its levels were higher in CAL compared CAH tissues (Fig. 7C). These data show that at least for some genes, we observed the same trend in protein expression.

Overall, our data suggests that CAL might develop due to disturbances in the M/SM healing rather than ME. We identified ECM remodeling and wound healing genes that CAL tissue lacked suggesting that these genes might be crucial for successful healing. We also showed that the expression level of these genes is highly dependent on the healing phase; therefore, the time of tissue collection is very critical when evaluating the potential use of a gene set as a biomarker.

## Discussions

Intestinal healing of surgically manipulated areas is a well-orchestrated process that involves different layers of the bowel wall. In case of surgical manipulation exerted by handling of the bowel but without transection, the ME layer has been the focus of many studies (Schneider et al. 2021, 2022; Stoffels et al. 2014). However, in case of anastomotic injury, which involves complete bowel wall transection, a transmural healing is required involving ME and M/SM. To the best of our knowledge the roles of the individual intestinal layers in CAH and CAL development have not been studied before. Herein, we provide a detailed transcriptional profiling of the M/SM and ME layers of the anastomotic tissues in CAL and CAH.

Our findings show that both layers follow the common stages of wound healing, namely inflammation, proliferation, and remodeling. Correlation analyses of the transcriptional profile of M/SM and ME at 6 h, 24 h and 72 h further indicated that these layers follow the wound healing stages mostly in synchronization. We observed that inflammation starts immediately after surgery in both layers and involves both innate and adaptive immune responses, which was also shown by van Helsdingen et al. recently (van Helsdingen et al. 2023) in a rat anastomosis model. Interestingly, inflammation appeared to decrease at 24 h and then significantly increase again at 72 h time point at M/SM layer. However, inflammatory responses were significantly upregulated at 24 h compared to 6 h time point and remained same at 72 h time point in the ME layer. This observation could be because the granulation tissue, which carries a large portion of the immune cells remained in the ME layer after separation.

Together with inflammation and defense responses, angiogenesis- and vasculature development-related pathways, including vascular endothelial growth factor (VEGF) production and regulation of VEGF production were significantly enriched in the ME and M/SM layers of the anastomosis in the early postoperative period. Even though, VEGF production is a downstream effect of HIF1 activation, which has been shown to play a particular role in anastomotic healing in a devascularisation-triggered CAL model (Strowitzki et al. 2021), we did not observe the upregulation of HIF1 target genes in the early stages (6 h and 24 h) of the healing process. This might be because HIF1 needs to accumulate and translocate to the nucleus to act as a transcription factor. Hence, HIF1 expression might be upregulated in the early stages of the healing in our model but its protein may not have reached the sufficient level to induce downstream gene expression. Therefore, regulation of angiogenesis might be HIF1-independent in the absence of hypoxia during anastomotic healing. Indeed, hypoxia is discussed as an important aspect in anastomotic healing deficiencies. Appropriate models, as the above mentioned devascularisation mode used by Strowitzki et al. (Strowitzki et al. 2021) are useful to study this particular aspect in anastomotic healing. However, conflicting data about a role of hypoxia in this model come from Shakhsheer et al., using a comparable segmental devascularisation together with colon anastomosis surgery in rats. They did not find any evidence for tissue hypoxia as a contributing factor in colon anastomotic leakage (Shakhsheer et al. 2017). In our study, which focused on healing and spontaneously occurring healing disturbances without any additional devascularisation procedure, we did not observe any difference in pathways directly-related to hypoxia, particularly no difference in HIF-1a target genes. Moreover, the gene expression analysis of HIF1 target genes did not show significant differences between CAL and CAH samples (Figure S9B). While these finding show that HIF-1a pathways are not relevant to CAL development in our model, they do not per se suggest that hypoxia is not involved in CAL development.

Nonetheless this early onset of angiogenic pathways in CAH tissues is interesting because, based on the histological evaluation of the anastomotic tissue, the neo-angiogenesis was reported to start 3 days after surgery in an anastomosis rat model (van Helsdingen et al. 2023). We also found a further enrichment of angiogenesis-related genes at 72 h within the M/SM layer. Interestingly, angiogenesis-associated genes were mostly different at 6 h and 72 h, indicating that early-stage induction of angiogenesis in the later stages might have different transcriptional pathways (Figure S9A).

Additionally, our results showed that the proliferation phase predominantly starts 24 h after surgery in both layers. Interestingly, GSEA showed both positive and negative regulation of proliferation. Analysis of M/SM layer showed a notable downregulation of various immune cell proliferation and upregulation of fibroblast proliferation (Figure S10). This suggests that regulation of proliferation is cell-type dependent but more comprehensive studies are required to described and quantify the complex immune cell landscape as well as fibroblast composition in CAL and CAH. Notably, we also observed a reduction in mesenchymal cell proliferation at 6 h, further supporting that regulation of proliferation might be cell-specific and can begin immediately after surgery. Interestingly, the proliferation phases are not fully synchronized between the M/SM and ME. While proliferation gene signatures become already downregulated at 72 h in the M/SM, they are still elevated and even higher at the 72 h time point compared to the 24 h in the ME. This might be also due to different cell populations present in both layers.

Due to high microbial load of the colon, sufficient and quick healing of anastomoses is very critical to prevent the organism from infections. Therefore, CAH needs to be completed in a short time (Morgan und Shogan 2022). In line with this, we observed enrichment of epithelial cell proliferation at 6 h and ECM remodeling at 72 h, which suggests that the transcriptional initiation of re-epithelization and tissue remodeling of the anastomotic wound might occur earlier than previously thought (Morgan und Shogan 2022). As a relevant example for the early onset of remodeling, we found collagen type I-III and V, which has been reported to be prominent in anastomotic healing are already highly induced at 72 h while other remodeling-associated collagens such as *Col16a1*, which supports strength but also cell invasion (Jensen et al. 2018), and *Col18a1*, which are *also* required for remodeling of the enteric nervous system (Nagy et al. 2018), are also induced.

Our results indicate that CAH is a very dynamic process already within the first 72 h. Whilst different wound healing stages are sequentially initiated, there is a substantial overlap between these stages in both layers. As our study did not include time points after 72 h, it remains unclear when these phases will be resolved. A recent finding indicated that the inflammation was still present 7 days after surgery (van Helsdingen et al. 2023), which is longer than previously thought (Morgan und Shogan 2022). Overall, our data indicate that the wound healing phases are transcriptionally overlapping and not strongly separated as indicated in a scheme shown in a recent study (Winter et al. 2023). Furthermore, our study shows that the first 72 h after surgery, which has been previously referred to as “inflammatory phase” (Winter et al. 2023), involves elements of all wound healing stages. Future studies are required to clarify if these quick transcriptional changes correspond to changes in protein levels and cellular compositions.

An important observation in this study is that on the transcriptional level, M/SM, but not ME, differs between CAL and CAH. In line with previous research we observed that M/SM of CAL tissues had reduction in ECM remodeling- and collagen-associated pathways (Shogan et al. 2016). In line with the transcriptomics data, we also observed lower levels of collagen in the tissues with CAL on protein level. This finding supports the observation of Shogan et al where devascularization-based CAL rat model had lower collagen content compared to properly healed anastomosis (Shogan et al. 2016). Additionally, our data shows that CAL mechanisms may not be limited to these pathways as we observed changes in various other processes such as cell migration and adhesion. However, since this analysis is based on tissues that already had leakage, it remains unknown whether these differences were the cause of CAL, or they were a result of other causative factors that might have taken place in earlier stages of the healing.

Nonetheless, here we provide a list of CAL-associated genes that might be used as leakage markers in mice in addition to other surrogate markers such as bursting pressure and histological examination of inflammatory cell infiltration, fibroblast activity and collagen deposition. Also, it is important to note that we identified the CAL pathways in the pre-operatively healthy intestine. For use in humans, other patient-related factors, such as the presence of cancer or intestinal inflammation, should be incorporated in future studies to validate if the genes we identified can serve as biomarkers for CAL. Due to newly developed digital rigid sigmoidoscope with the ability to biopsy for surgical rectal assessments (Lewis et al. 2021), the gene list we provide might be useful to identify perianastomotic mucosal CAL signatures in patients at risk to develop CAL. Also, proteins of some of these CAL–related genes, such as *Anxa1*, *Ccn1* and *Krt10*, have been previously detected in circulation and therefore might serve as blood markers for CAL. Although our study mainly focused on the transcriptional patterns some limited analyses on the condensed list of DEGs differing between CAL and CAH showed that their expression pattern follow the ones observed in the transcriptional analyses. This is particular true for the TGF-β decoy receptor Bambi (Onichtchouk et al. 1999), negatively regulating TGF-β signaling and partially also for IL-34, which both have been shown to contribute to wound healing and the related inflammation (Ehnert et al. 2023; Yang et al. 2017; Lin et al. 2015; Monteleone et al. 2022). As TGF-β signaling is required for mucosal healing (Beck et al. 2003) and tissue maintenance. It plays also an important role in barrier integrity in inflammatory bowel diseases (IBD) (Sommer et al. 2021), and a negative regulation, as it might occurs under higher Bambi expression, could be indeed a crucial trigger for CAL development. The role of IL-34 in wound healing, particular in intestinal healing is not well understood, but it is discussed as a modulator of IBD as well as a strong protective immune mediator in gastrointestinal graft-versus host disease (Zwicker et al. 2015; Rayasam et al. 2025). Their particular role and the question if one of these molecules can be also used as a potential biomarker for CAL or CAH must be studied in further longitudinal studies on local tissue and blood circulation levels.

## Conclusions

In conclusion, our study establishes a transcriptional framework of CAH and CAL in mice and provides an inventory of genes associated with various pathways related to both processes. Our findings provide first insight into longitudinal gene expression profiles with suggestions for mediators potentially worth to be further investigated as predictors of CAL. Furthermore, our data are a valuable toolbox useful for future CAH and CAL studies indicating appropriate gene sets to be studied in different phases of anastomotic inflammation and healing.

## Supplementary Information

Additional file 1: Table 1: Table of 31 of the 569 DEGs between CAL and CAH at 72 h based on a log2 value of at least -1 or +1.5, respectivly and with and adjusted p-value of less than 0.05. The genes were considered for further analysis of protein expression. Figure S1: **A** Anastomotic complication scoring (ACS) system used in this study. **B** Representative figures for each score. Gray arrow line shows the anastomotic region without defect and red arrow line shows anastomosis with different severity of defect.

Additional file 2: Figure S1: **A** Anastomotic complication scoring (ACS) system used in this study. **B** Representative figures for each score. Gray arrow line shows the anastomotic region without defect and red arrow line shows anastomosis with different severity of defect.

Additional file 3: Figure S2: Step-by-step layer separation process. **A** Anastomotic region is identified. **B** Attachments are removed. **C** Defect is identified, and ACS is determined. **D** Anastomotic tissue is collected. **E** Tissue is pinned, and mucosa/submucosa layer is separated from the muscularis externa with forceps. **F** Layers are separated. Red arrows show the anastomotic region.

Additional file 4: Figure S3 **A** Enrichment plots of Type I IFN and IFNγ response in the M/SM layer of the anastomotic tissue at 6h compared to naive controls. **B** Significantly enriched gene ontology terms related to vascularisation pathways in the M/SM tissue of anastomosis at 6h after surgery compared to naïve conditions. **C** Heatmap of HIF-1a-target genes expressed in the M/SM layer of anastomotic tissue at 6h, 24h and 72h and naïve animals based on GSEA. **D** Significantly enriched gene ontology terms related to vascularisation pathways in the ME tissue of anastomosis at 6h after surgery compared to naïve conditions. **E** Heatmap of HIF-1α-target genes expressed in the ME layer of anastomotic tissue at 6h, 24h and 72h and naïve animals based on GSEA.

Additional file 5: Figure S4: Gene expression profile of the ME layer of the anastomotic tissue at 6h compared to naive controls. **A** Volcano plot of DEGs in the ME layer of anastomotic tissue collected at 6h compared to naive tissue (red and blue shows up-/downregulated genes, respectively). **B** Selected significantly enriched gene sets in the ME of anastomosis at 6h time point compared to the naive tissue based on GSEA.

Additional file 6: Figure S5: Selected significantly enriched pathways in the M/SM of anastomosis at 6h time point compared to the naive tissue based on GSEA.

Additional file 7: Figure S6: Gene expression profile of the ME layer of the anastomotic tissue at 24h compared to 6h. **A** Volcano plot of DEGs in the ME layer of anastomotic tissue collected at 24h compared to 6h. (red and blue shows up-/downregulated genes, respectively). **B** Selected significantly enriched pathways in the ME at 24h time point compared to 6h.

Additional file 8: Figure S7: Gene expression profile of the ME layer of the anastomotic tissue at 72h compared to 24h. **A** Volcano plot of DEGs in the ME layer of anastomotic tissue collected at 72h compared to 24h. (red and blue shows up-/downregulated genes, respectively) **B** Correlation plot based on the Wald statistics of the genes shared between M/SM and ME layer at 72h after surgery compared to 24h. **C** Selected significantly enriched pathways in the ME layer of anastomotic tissue at 72h compared to 24h time point based on GSEA. **D** Heatmap showing the expression pattern of ECM modelling-related gene expression in the ME layer of naive and anastomotic samples collected at 6h, 24h and 72h. **E** Heatmap showing the expression pattern of all the genes coding for collagens and MMPs detected in the ME of naive and anastomotic samples collected at 6h, 24h and 72h.

Additional file 9: Figure S8: Enrichment plots of extracellular matrix (cellular component) in the M/SM layer of CAL tissue compared to CAH.

Additional file 10: Figure S9: **A** Venn diagram showing the number of angiogenesis-related genes specific to and shared between 6h and 72h time point. **B** Heatmap of HIF-1α-target genes expressed in the M/SM layer of CAH and CAL anastomosis tissue 72h after surgery based on GSEA.

Additional file 11: Figure S10: Enrichment plot of fibroblast, leukocyte, and lymphocyte proliferation in the M/SM layer of the anastomotic tissue collected at 24h compared to 6h.

Additional file 12: Figure S11: **A** Experimental setup scheme. Mice underwent anastomosis surgery at time point 0. 72h hours later anastomosis were prepared, embedded in paraffin, sectioned and underwent Masson Goldner Trichome (MGT) staining allowing collagen content quantification (green areas). **B** Representative pictures of MGT stainings from CAH and CAL anastomoses used for collagen content quantification shown in figure 7. Scale bar in the enlarged image section = 50µm.

## Data Availability

The data supporting this study’s findings are available on request from the corresponding author. Sequencing data will be available via https://www.ncbi.nlm.nih.gov/geo/after acceptance of the paper.

## Abbreviations

ACS: Anastomotic complication score
CAH: Colon anastomotic healing
CAL: Colon anastomotic leakage
ECM: Extracellular matrix
GO: Gene ontology
GSEA: Gene set enrichment analysis
ME: Muscularis externa
MMPs: Matrix metalloproteinases
M/SM: Mucosa and submucosa
