# Supplementary material for: Layer-specific molecular signatures of colon anastomotic healing and leakage in mice
Source: Mol Med. 2025 Apr 1;31:124. doi: 10.1186/s10020-025-01167-9 (PMC11959837; doi:10.1186/s10020-025-01167-9)
Supplement: Supplementary file 1 — Additional file 1: Table 1: Table of 31 of the 569 DEGs between CAL and CAH at 72 h based on a log2 value of at least -1 or +1.5, respectivly and with and adjusted p-value of less than 0.05. The genes were considered for further analysis of protein expression. Figure S1: A Anastomotic complication scoring (ACS) system used in this study. B Representative figures for each score. Gray arrow line shows the anastomotic region without defect and red arrow line shows anastomosis with different severity of defect. [file 10020_2025_1167_MOESM1_ESM.pdf]

Supplemental table 1

Target	log2FoldChange	padj	Protein Name
Kng2	-4,539229159	0,040460051	kininogen 2
Spp1	-1,770008856	0,01013908	Osteopontin
Adm	-1,444009024	0,000444068	adrenomedullin
Adamtsl1	-1,368945297	0,025234883	ADAMTS-like protein 1
Jaml	-1,312253532	0,001796225	Junction Adhesion Molecule Like
Ccdc80	-1,303581404	0,000110546	Coiled-Coil Domain Containing 80
Camsap2	-1,256065435	0,003344439	Calmodulin Regulated Spectrin Associated Protein Family Member 2
C4b	-1,156928985	0,019694041	Complement C4B
Aebp1	-1,135015614	0,009107853	AE Binding Protein 1
Clec4n	-1,089384877	0,026146088	C-type lectin domain-containing protein
S100a4	-1,088834258	0,000311583	S100 Calcium Binding Protein A4
Timp1	-1,081480066	0,002044254	TIMP Metallopeptidase Inhibitor 1
Aif1	-1,048662931	0,004333235	Allograft Inflammatory Factor 1
Il34	-1,038009584	0,017964588	Interleukin 34
Akap12	-1,016257808	0,025234883	A-Kinase Anchoring Protein 12
Txnip	1,622836213	1,31E-08	Thioredoxin-interacting protein
Cox7c	1,657512426	7,54E-24	Cytochrome C Oxidase Subunit 7C
Foxk1	1,661535087	0,000423537	Forkhead Box K1
Car9	1,893107343	3,64E-07	carbonic anhydrase 9
Cox17	1,904250211	5,56E-11	Cytochrome C Oxidase Copper Chaperone *COX17*
Arpin	1,904261884	1,36E-08	Arpin
Trip10	2,081998267	1,00E-05	Thyroid Hormone Receptor Interactor 10
Ctnnd1	2,468393682	1,00E-05	Catenin Delta 1
Ldb1	2,572788725	8,05E-05	LIM Domain Binding 1
Dnaja4	2,749303723	0,02990777	DnaJ Heat Shock Protein Family (Hsp40) Member A4
Gtse1	2,983950589	0,017964588	G2 and S phase-expressed protein 1
Bambi	3,113260582	6,70E-05	BMP And Activin Membrane Bound Inhibitor
Clcn2	4,385038187	0,026694343	Chloride Voltage-Gated Channel 2
Nphs1	4,441678534	1,71E-05	NPHS1 Adhesion Molecule, Nephrin
Ccn1	4,970083463	0,020563538	Cellular Communication Network Factor 1
Btnl2	5,176064196	0,00638116	Butyrophilin Like 2
