# Supplementary material for: Layer-specific molecular signatures of colon anastomotic healing and leakage in mice
Source: Mol Med. 2025 Apr 1;31:124. doi: 10.1186/s10020-025-01167-9 (PMC11959837; doi:10.1186/s10020-025-01167-9)
Supplement: Supplementary file 2 — Additional file 2: Figure S1: A Anastomotic complication scoring (ACS) system used in this study. B Representative figures for each score. Gray arrow line shows the anastomotic region without defect and red arrow line shows anastomosis with different severity of defect. [file 10020_2025_1167_MOESM2_ESM.pdf]

Supplementary Figure 1

A)

Score	Anastomotic Complication
**0**	No adhesions or abnormalities
**1**	Adhesion to fat pad, clean anastomosis underneath
**2**	Adhesion to intestinal loop, abdominal wall, or another organ
**3**	Anastomotic defect found underneath adhesions,<br>no other abnormalities
**4**	Signs of possible contamination (e.g., small abscesses)
**5**	Clear anastomotic complication; spread of pus,<br>obstruction at anastomosis,<br>sign of peritonitis
**6**	Fecal peritonitis/Death due to peritonitis

B) Representative Images for Each ACS

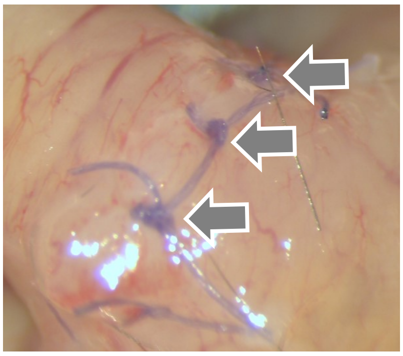

No adhesions or abnormalities, ACS = 0

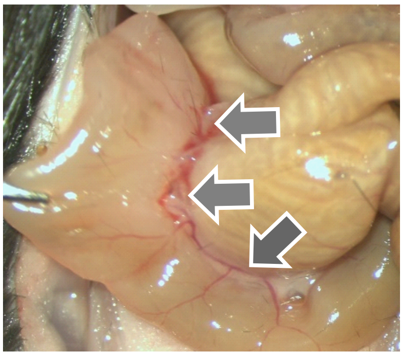

Adhesions around anastomosis without defect, ACS = 1 or 2

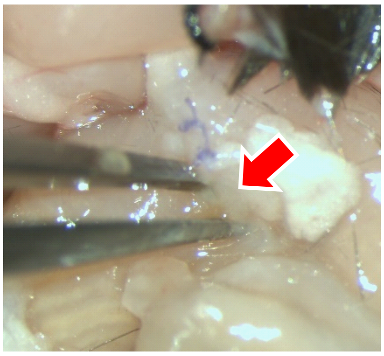

Adhesions around anastomosis with defect, ACS = 3

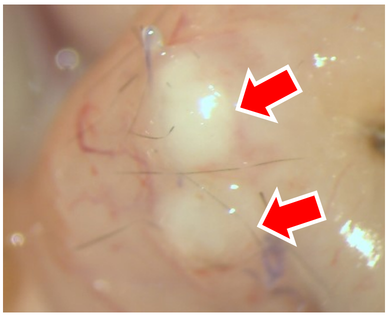

Abscess formation, ACS = 4

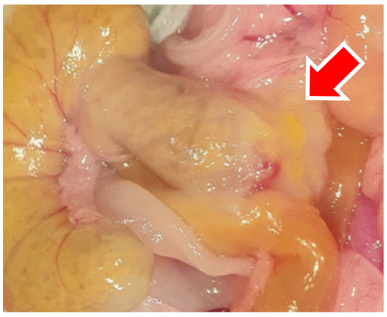

Sign of peritonitis, ACS = 5

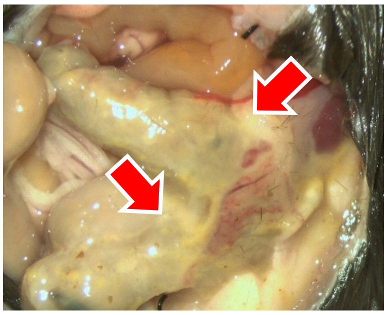

Fecal peritonitis, ACS = 6
