# Supplementary figures and images for: Layer-specific molecular signatures of colon anastomotic healing and leakage in mice
Source: Mol Med. 2025 Apr 1;31:124. doi: 10.1186/s10020-025-01167-9 (PMC11959837; doi:10.1186/s10020-025-01167-9)

Supplementary Figure 2

A)

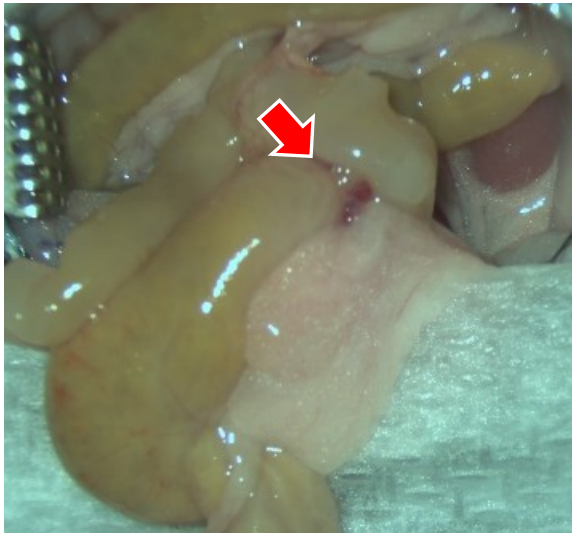

B)

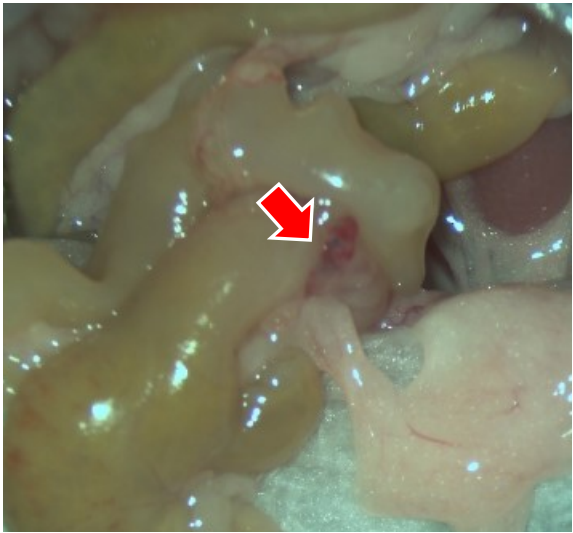

C)

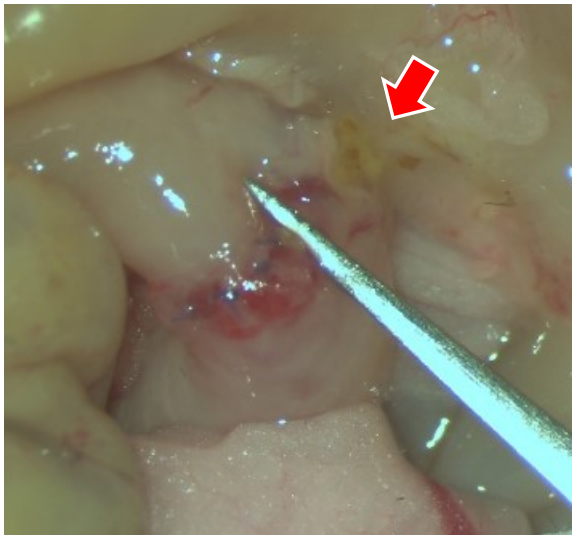

D)

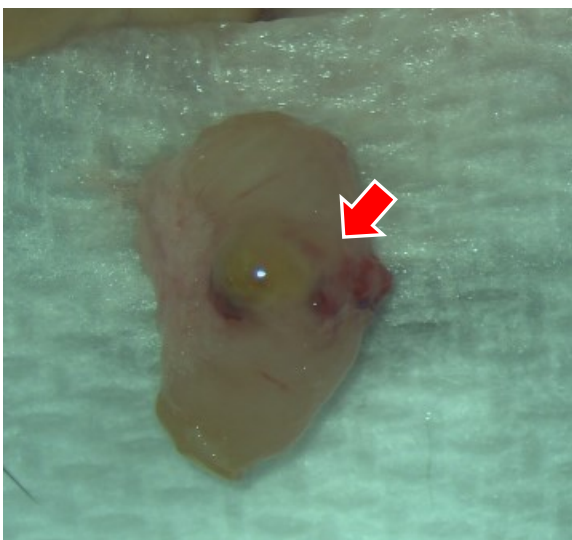

E)

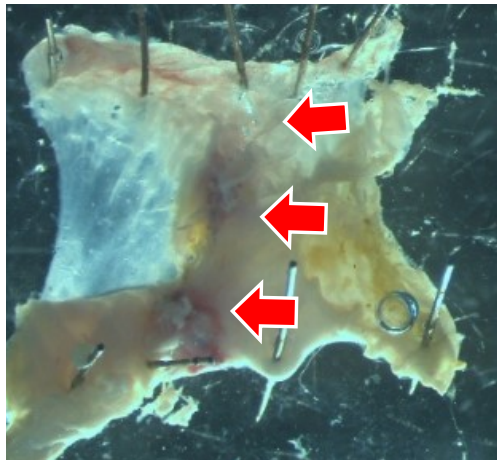

F)

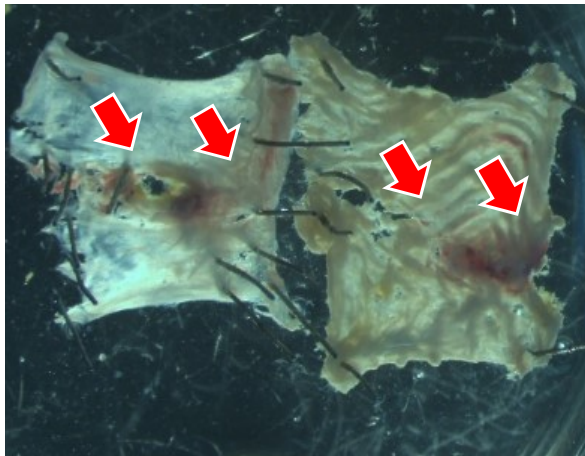

Supplement: Supplementary file 3 — Additional file 3: Figure S2: Step-by-step layer separation process. A Anastomotic region is identified. B Attachments are removed. C Defect is identified, and ACS is determined. D Anastomotic tissue is collected. E Tissue is pinned, and mucosa/submucosa layer is separated from the muscularis externa with forceps. F Layers are separated. Red arrows show the anastomotic region. [file 10020_2025_1167_MOESM3_ESM.pdf]

Supplementary Figure 3

A

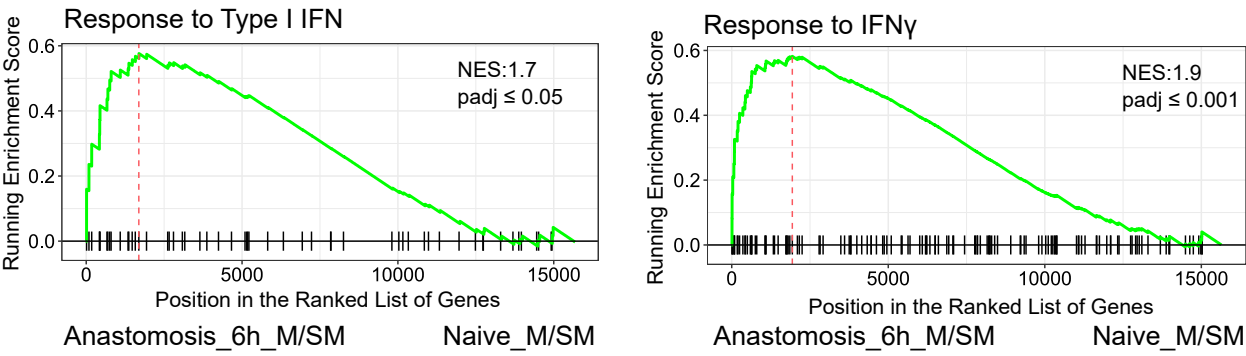

B

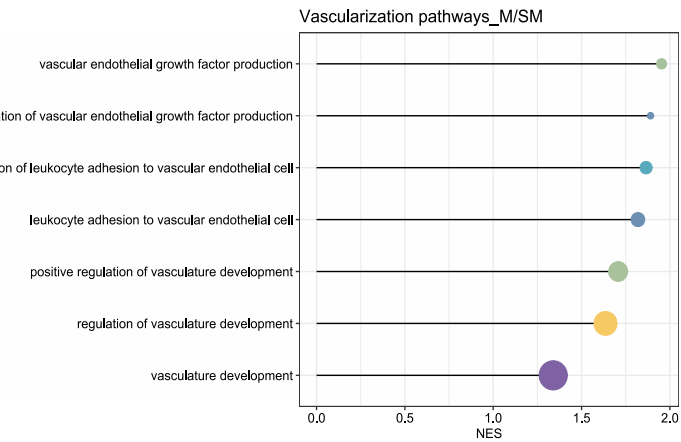

C

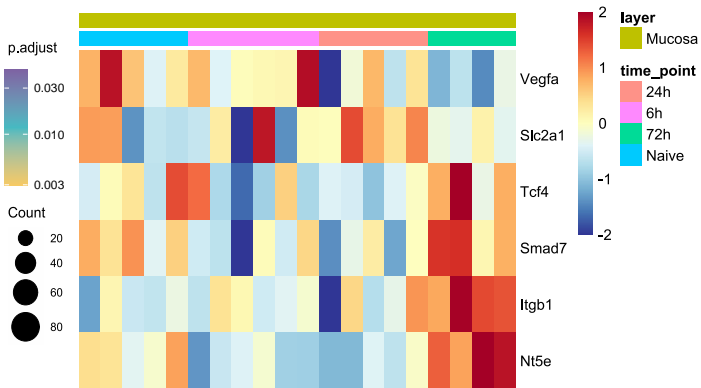

D

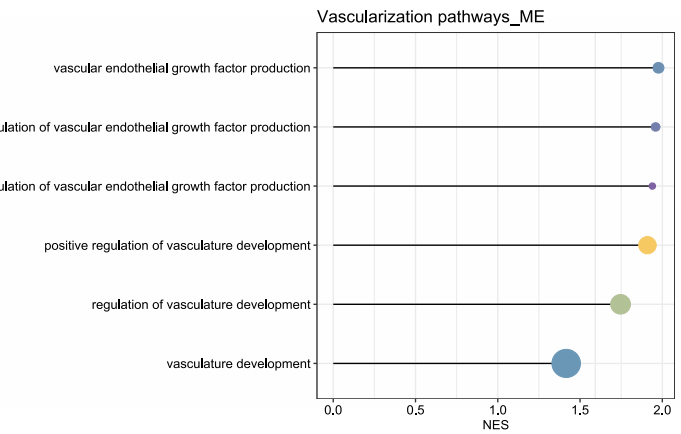

E

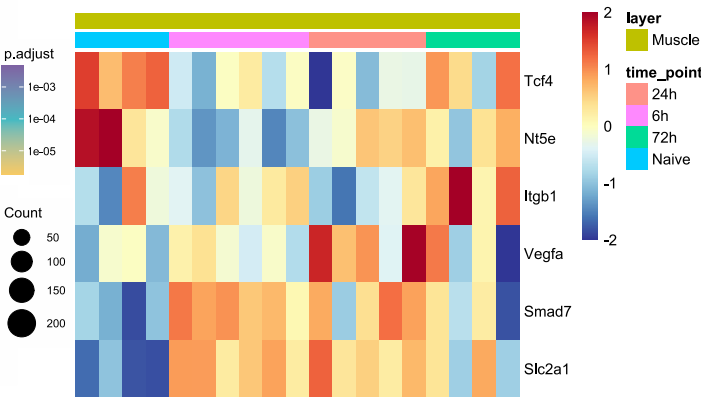

Supplement: Supplementary file 4 — Additional file 4: Figure S3 A Enrichment plots of Type I IFN and IFNγ response in the M/SM layer of the anastomotic tissue at 6h compared to naive controls. B Significantly enriched gene ontology terms related to vascularisation pathways in the M/SM tissue of anastomosis at 6h after surgery compared to naïve conditions. C Heatmap of HIF-1a-target genes expressed in the M/SM layer of anastomotic tissue at 6h, 24h and 72h and naïve animals based on GSEA. D Significantly enriched gene ontology terms related to vascularisation pathways in the ME tissue of anastomosis at 6h after surgery compared to naïve conditions. E Heatmap of HIF-1α-target genes expressed in the ME layer of anastomotic tissue at 6h, 24h and 72h and naïve animals based on GSEA. [file 10020_2025_1167_MOESM4_ESM.pdf]

Supplementary Figure 6

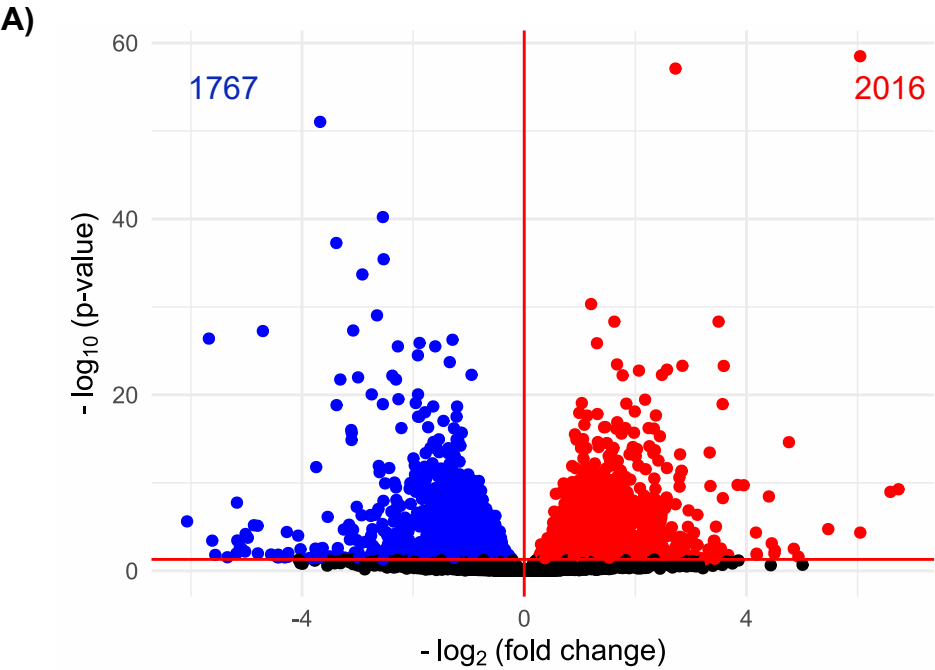

B) Enriched Gene Sets in the ME of Anastomosis at 24h vs 6h

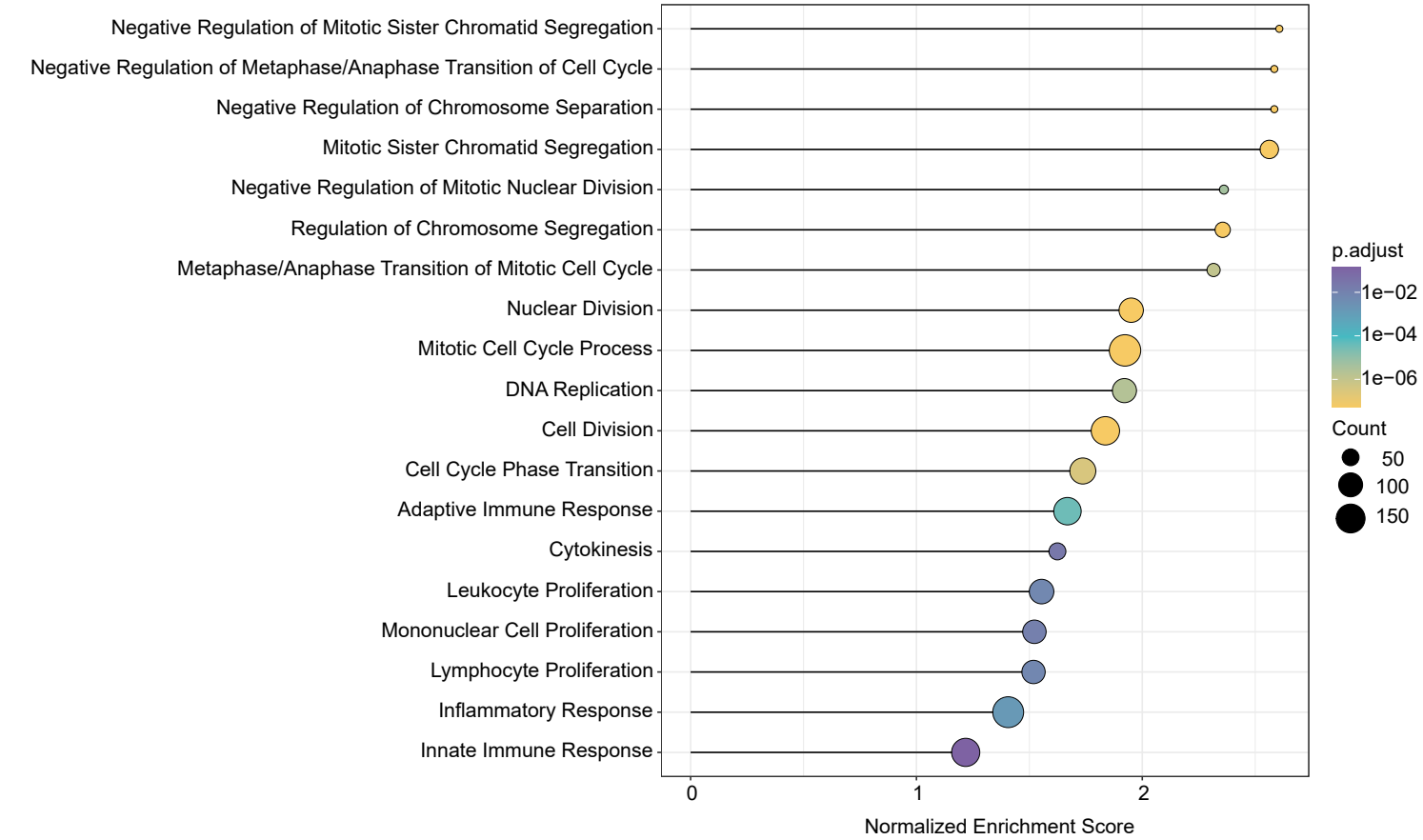

Supplement: Supplementary file 7 — Additional file 7: Figure S6: Gene expression profile of the ME layer of the anastomotic tissue at 24h compared to 6h. A Volcano plot of DEGs in the ME layer of anastomotic tissue collected at 24h compared to 6h. (red and blue shows up-/downregulated genes, respectively). B Selected significantly enriched pathways in the ME at 24h time point compared to 6h. [file 10020_2025_1167_MOESM7_ESM.pdf]

Supplementary Figure 8

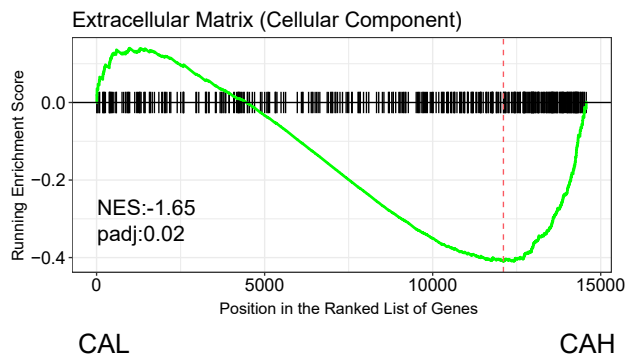

Supplement: Supplementary file 9 — Additional file 9: Figure S8: Enrichment plots of extracellular matrix (cellular component) in the M/SM layer of CAL tissue compared to CAH. [file 10020_2025_1167_MOESM9_ESM.pdf]

# Supplementary Figure 9

**A** Number of angiogenesis-associated genes

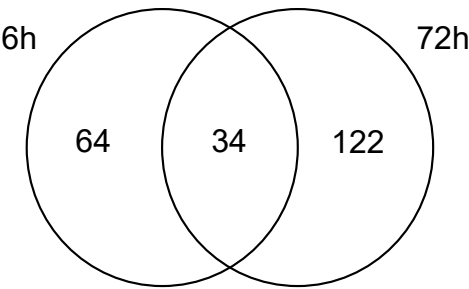

**B**

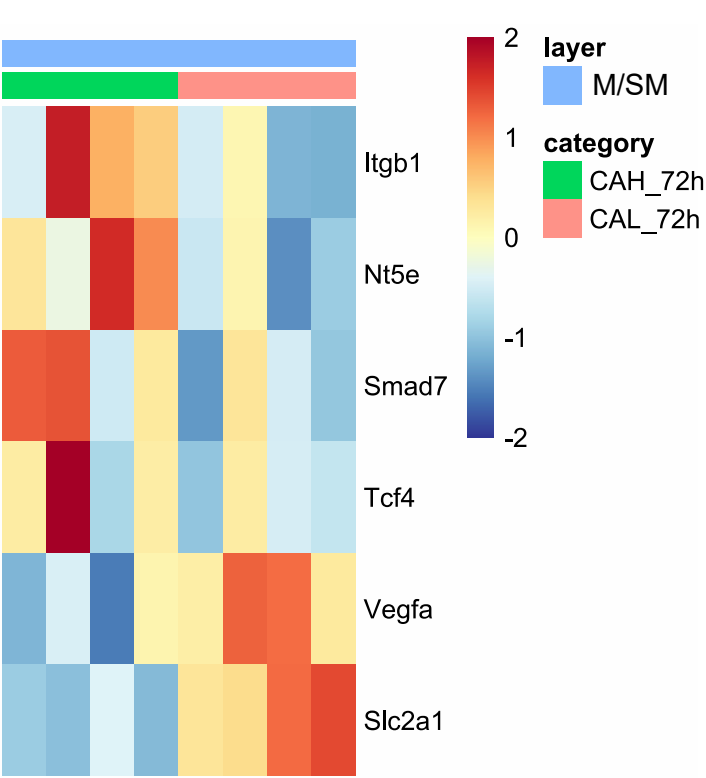

Supplement: Supplementary file 10 — Additional file 10: Figure S9: A Venn diagram showing the number of angiogenesis-related genes specific to and shared between 6h and 72h time point. B Heatmap of HIF-1α-target genes expressed in the M/SM layer of CAH and CAL anastomosis tissue 72h after surgery based on GSEA. [file 10020_2025_1167_MOESM10_ESM.pdf]

Supplementary Figure 10

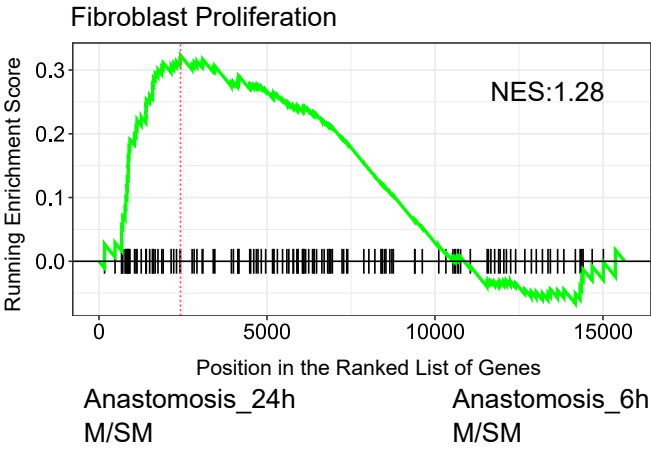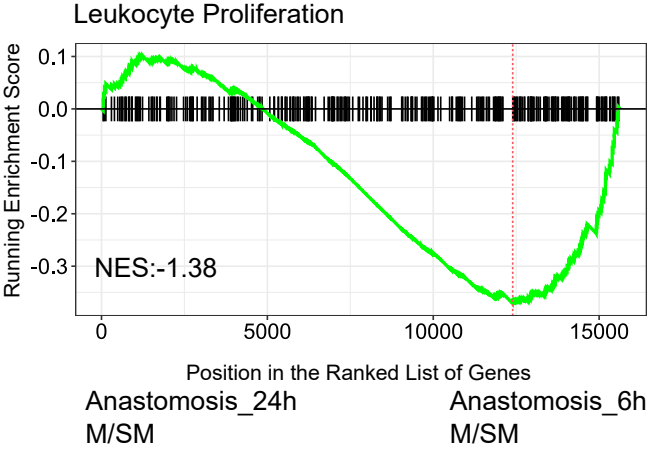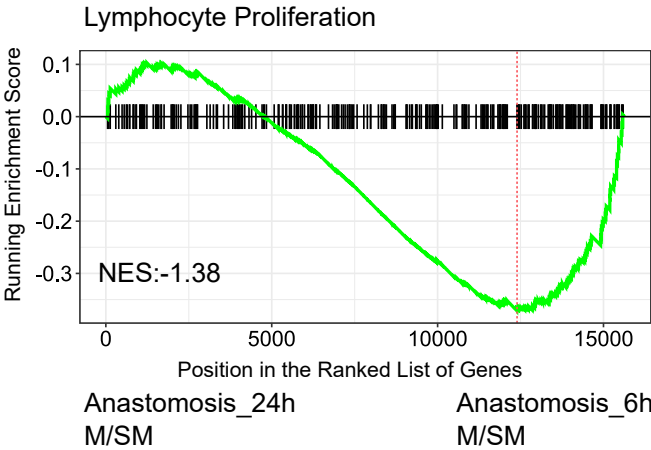

Supplement: Supplementary file 11 — Additional file 11: Figure S10: Enrichment plot of fibroblast, leukocyte, and lymphocyte proliferation in the M/SM layer of the anastomotic tissue collected at 24h compared to 6h. [file 10020_2025_1167_MOESM11_ESM.pdf]

# Supplementary Figure 11

A)

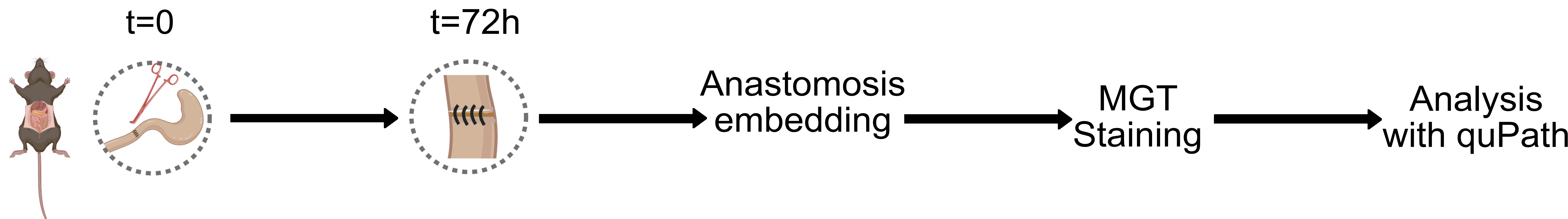

B)

**CAH**

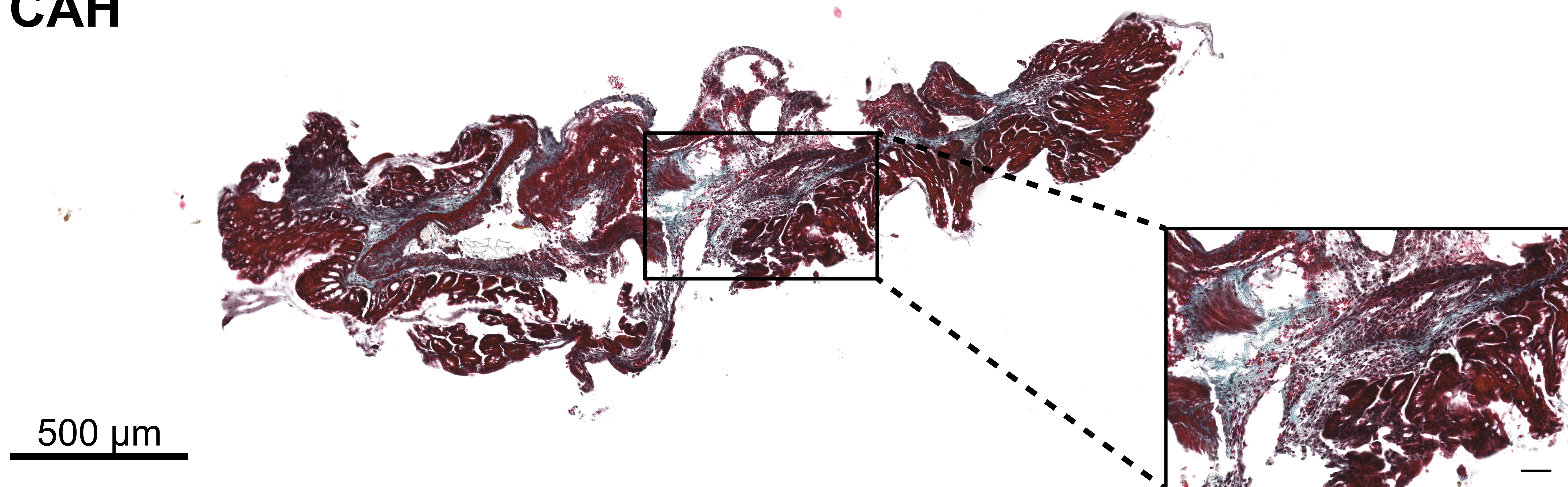

**CAL**

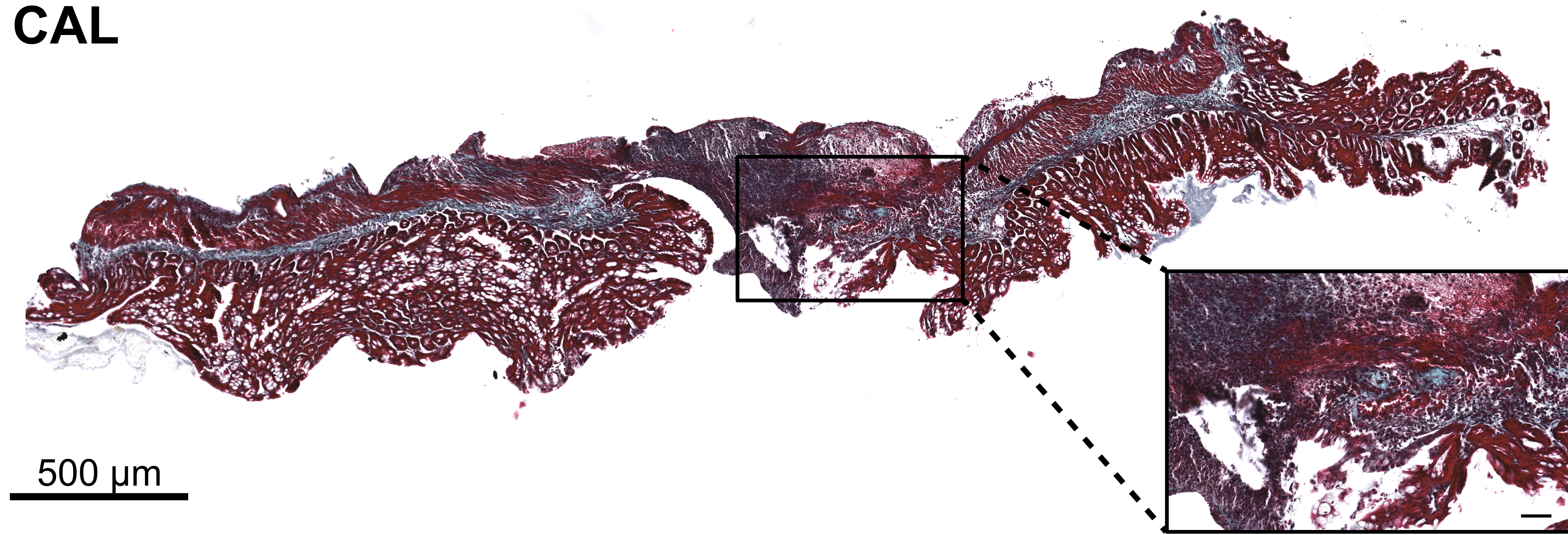

Supplement: Supplementary file 12 — Additional file 12: Figure S11: A Experimental setup scheme. Mice underwent anastomosis surgery at time point 0. 72h hours later anastomosis were prepared, embedded in paraffin, sectioned and underwent Masson Goldner Trichome (MGT) staining allowing collagen content quantification (green areas). B Representative pictures of MGT stainings from CAH and CAL anastomoses used for collagen content quantification shown in figure 7. Scale bar in the enlarged image section = 50µm. [file 10020_2025_1167_MOESM12_ESM.pdf]
