# Supplementary material for: Layer-specific molecular signatures of colon anastomotic healing and leakage in mice
Source: Mol Med. 2025 Apr 1;31:124. doi: 10.1186/s10020-025-01167-9 (PMC11959837; doi:10.1186/s10020-025-01167-9)

Supplementary Figure 4

A)

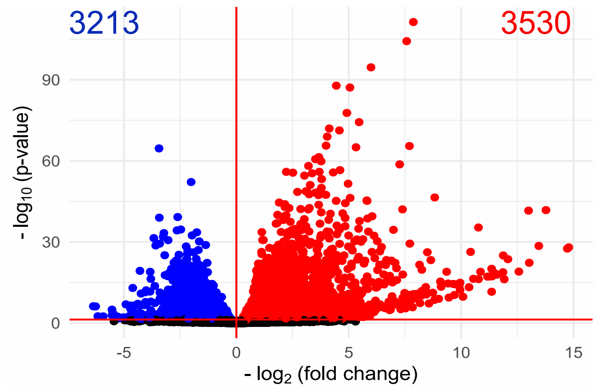

B) Enriched Gene Sets in ME of Anastomotic Tissue at 6h compared to Naive Controls

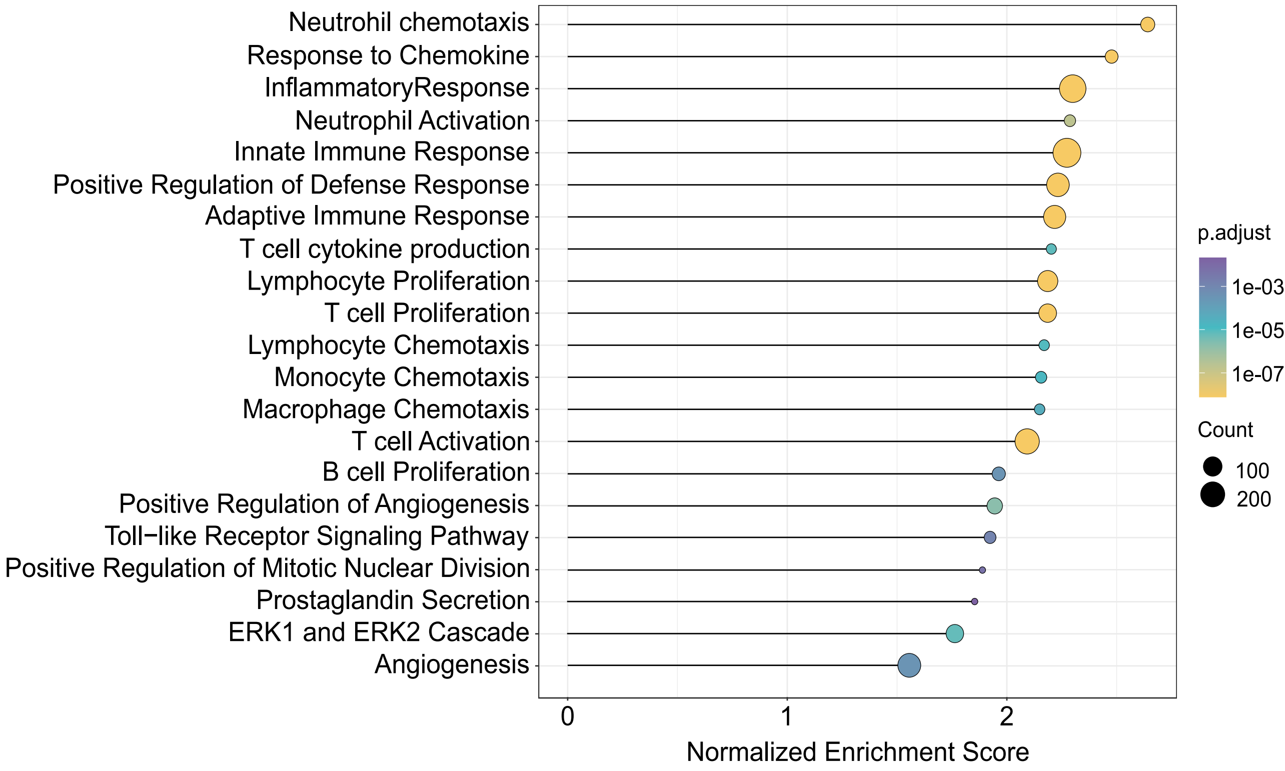

Supplement: Supplementary file 5 — Additional file 5: Figure S4: Gene expression profile of the ME layer of the anastomotic tissue at 6h compared to naive controls. A Volcano plot of DEGs in the ME layer of anastomotic tissue collected at 6h compared to naive tissue (red and blue shows up-/downregulated genes, respectively). B Selected significantly enriched gene sets in the ME of anastomosis at 6h time point compared to the naive tissue based on GSEA. [file 10020_2025_1167_MOESM5_ESM.pdf]
