# Supplementary material for: Layer-specific molecular signatures of colon anastomotic healing and leakage in mice
Source: Mol Med. 2025 Apr 1;31:124. doi: 10.1186/s10020-025-01167-9 (PMC11959837; doi:10.1186/s10020-025-01167-9)

Supplementary Figure 5

Enriched Gene Sets in M/SM of Anastomotic Tissue at 24h Compared to Naive Tissue

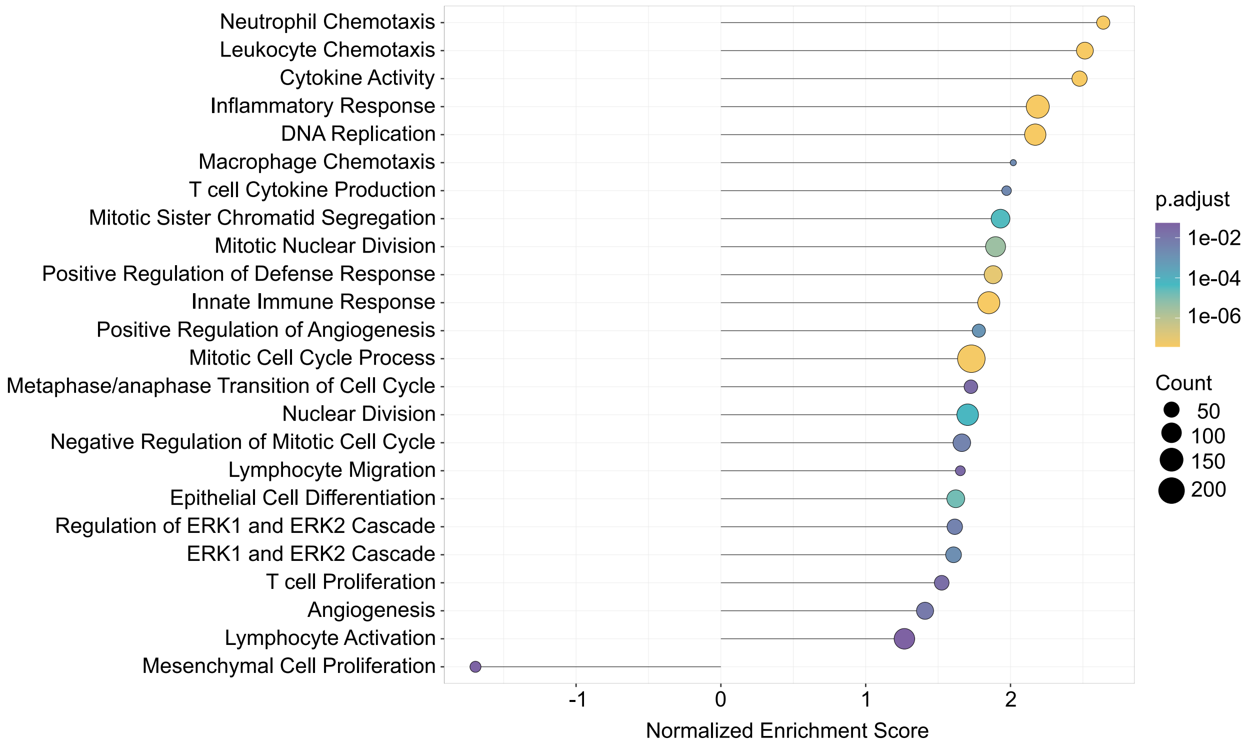

Supplement: Supplementary file 6 — Additional file 6: Figure S5: Selected significantly enriched pathways in the M/SM of anastomosis at 6h time point compared to the naive tissue based on GSEA. [file 10020_2025_1167_MOESM6_ESM.pdf]
