# Supplementary material for: Layer-specific molecular signatures of colon anastomotic healing and leakage in mice
Source: Mol Med. 2025 Apr 1;31:124. doi: 10.1186/s10020-025-01167-9 (PMC11959837; doi:10.1186/s10020-025-01167-9)

Supplementary Figure 7

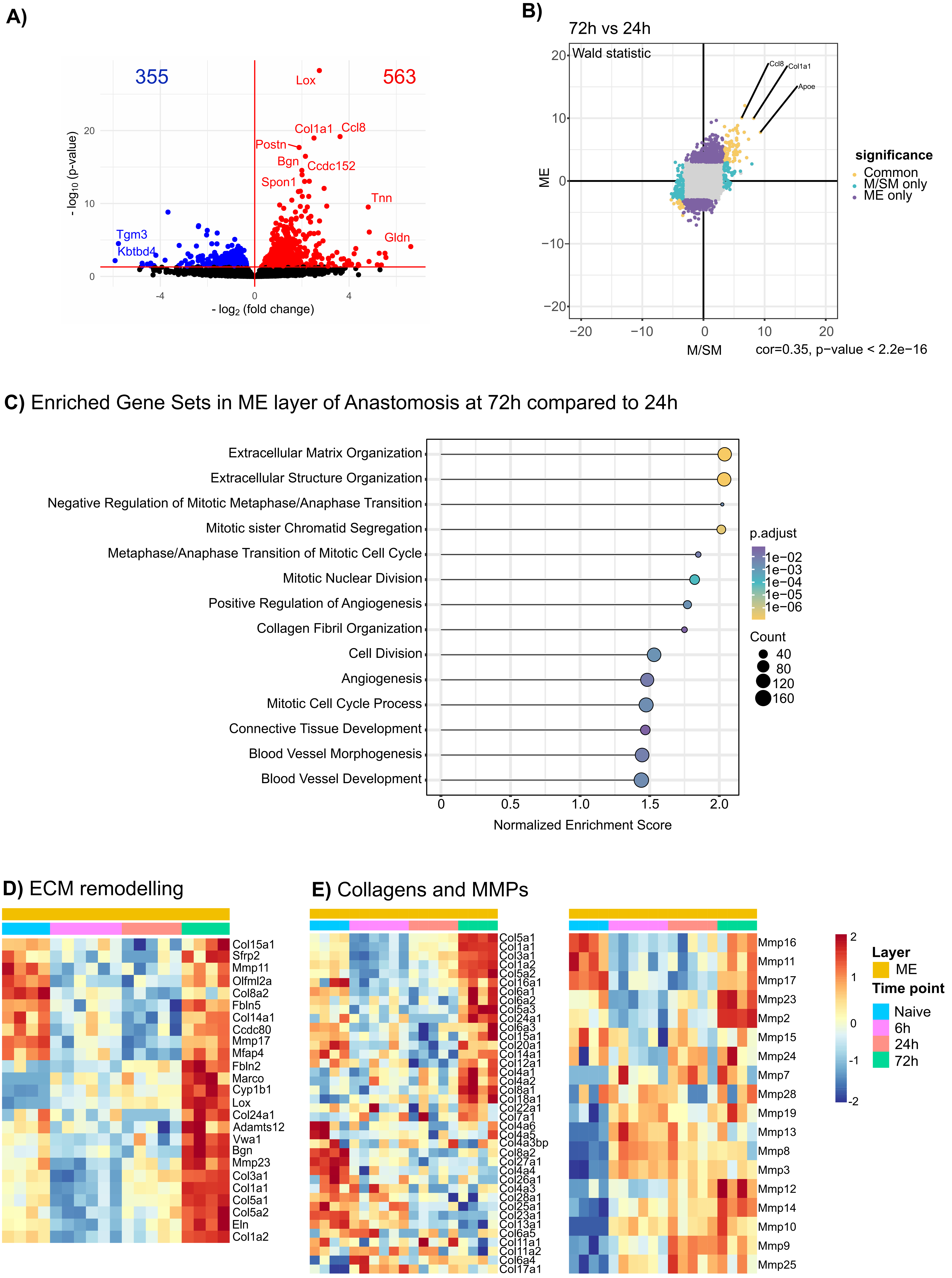

Supplement: Supplementary file 8 — Additional file 8: Figure S7: Gene expression profile of the ME layer of the anastomotic tissue at 72h compared to 24h. A Volcano plot of DEGs in the ME layer of anastomotic tissue collected at 72h compared to 24h. (red and blue shows up-/downregulated genes, respectively) B Correlation plot based on the Wald statistics of the genes shared between M/SM and ME layer at 72h after surgery compared to 24h. C Selected significantly enriched pathways in the ME layer of anastomotic tissue at 72h compared to 24h time point based on GSEA. D Heatmap showing the expression pattern of ECM modelling-related gene expression in the ME layer of naive and anastomotic samples collected at 6h, 24h and 72h. E Heatmap showing the expression pattern of all the genes coding for collagens and MMPs detected in the ME of naive and anastomotic samples collected at 6h, 24h and 72h. [file 10020_2025_1167_MOESM8_ESM.pdf]
